# Differential prognostic impact of myelodysplasia-related gene mutations in a European cohort of 4978 intensively treated AML patients

**DOI:** 10.1038/s41375-025-02781-6

**Published:** 2025-10-27

**Authors:** Marius Bill, Jan-Niklas Eckardt, Konstanze Döhner, Maximillian-Alexander Röhnert, Christian Rausch, Klaus H. Metzeler, Karsten Spiekermann, Sebastian Stasik, Alexander A. Wurm, Tim Sauer, Sebastian Scholl, Ulf Schnetzke, Andreas Hochhaus, Martina Crysandt, Tim H. Brümmendorf, Utz Krug, Bernhard Wörmann, Hermann Einsele, Wolfgang Hiddemann, Dennis Görlich, Cristina Sauerland, Björn Steffen, Andreas Neubauer, Andreas Burchert, Kerstin Schäfer-Eckart, Wolfgang E. Berdel, Christoph Schliemann, Stefan W. Krause, Mathias Hänel, Maher Hanoun, Martin Kaufmann, Lars Fransecky, Jan Braess, Johannes Schetelig, Jan Moritz Middeke, Lars Bullinger, Michael Heuser, Felicitas Thol, Hubert Serve, Claudia D. Baldus, Uwe Platzbecker, Carsten Müller-Tidow, Jan Válka, Jiří Šrámek, Barbora Weinbergerova, Jiri Mayer, Pierre-Yves Dumas, Sarah Bertoli, Eric Delabesse, Christian Récher, Arnaud Pigneux, Tobias Herold, Arnold Ganser, Hartmut Döhner, Martin Bornhäuser, Christian Thiede, Christoph Röllig

**Affiliations:** 1 Department of Internal Medicine I, University Hospital TU Dresden; Fetscherstraße 74; 01307 Dresden, Saxony, Germany; 2 Mildred Scheel Early Career Center, Medical Clinic and Policlinic I, University Hospital of the Technical University Dresden, Dresden, Germany; 3 National Center for Tumor Diseases Dresden (NCT/UCC), Medical Faculty and University Hospital Carl Gustav Carus, Technical University Dresden, Dresden, Germany; 4 German Cancer Consortium (DKTK), Dresden, Germany; 5 Department of Internal Medicine III, Ulm University Hospital, Ulm, Germany; 6 Laboratory for Leukemia Diagnostics, Department of Medicine III, LMU University Hospital, LMU Munich, Munich, Germany; 7 Department of Hematology, Cell Therapy, Hemostaseology and Infectious Diseases, University Hospital Leipzig, Leipzig, Germany; 8 German Cancer Research Center (DKFZ) and Medical Clinic V, University Hospital Heidelberg, Heidelberg, Germany; 9 Klinik für Innere Medizin II, Jena University Hospital, Jena, Germany; 10 Department of Hematology, Oncology, Hemostaseology, and Cell Therapy, University Hospital RWTH Aachen, Aachen, Germany; 11 DKMS Collection Center, Cologne, Germany; 12 Department of Hematology, Oncology and Tumor Immunology, Charité – Universitätsmedizin Berlin, Corporate Member of Freie Universität Berlin and Humboldt-Universität zu Berlin, Berlin, Germany; 13 Department of Internal Medicine II, University Hospital Würzburg, Würzburg, Germany; 14 Institute for Biostatistics and Clinical Research, University Muenster, Münster, Germany; 15 Medical Clinic II, University Hospital Frankfurt, Frankfurt (Main), Germany; 16 Department of Hematology, Oncology and Immunology, Philipps-University-Marburg, Marburg, Germany; 17 Department of Internal Medicine V, Paracelsus Medizinische Privatuniversität and University Hospital Nuremberg, Nuremberg, Germany; 18 Department of Medicine A, University Hospital Münster, Münster, Germany; 19 Department of Medicine 5, Uniklinikum Erlangen, Erlangen, Germany; 20 Department of Internal Medicine III, Klinikum Chemnitz gGmbH, Chemnitz, Germany; 21 Department of Hematology and Stem Cell Transplantation, University Hospital Essen, Essen, Germany; 22 Department of Hematology, Oncology and Palliative Care, Robert-Bosch-Hospital, Stuttgart, Germany; 23 Department of Internal Medicine, University Hospital Kiel, Kiel, Germany; 24 Hospital Barmherzige Brueder Regensburg, Regensburg, Germany; 25 Department of Internal Medicine IV, Hematology, Oncology, University Hospital Halle/Saale, Halle, Germany; 26 Department of Hematology, Hemostasis, Oncology & Stem Cell Transplantation, Hannover Medical School, Hannover, Germany; 27 Institute of Hematology and Blood Transfusion, Prague, Czech Republic; 28 Department of Haematology and Oncology, University Hospital Pilsen, Pilsen, Czech Republic; 29 Histology and Embryology Department, Faculty of Medicine in Pilsen, Charles University, Prague, Czech Republic; 30 Department of Internal Medicine, Hematology and Oncology, Masaryk University Hospital, Brno, Czech Republic; 31 CHU Bordeaux, Service d’Hématologie Clinique et de Thérapie Cellulaire, Bordeaux, France; 32 Centre Hospitalier Universitaire de Toulouse, Institut Universitaire du Cancer de Toulouse Oncopole, Université de Toulouse, Toulouse, France; 33 Department of Hematology and Oncology, Munich Hospital Bogenhausen, Munich Municipal Hospital Group, Munich, Germany

**Keywords:** Risk factors, Prognosis, Translational research

## Abstract

In the European LeukemiaNet (ELN) 2022 recommendations, myelodysplasia-related (MR) gene mutations were classified as a novel adverse prognostic category for intensively treated acute myeloid leukemia (AML). To assess the prognostic impact of individual MR genes within the ELN, clinical, cytogenetic, and molecular data from 4,978 intensively treated AML patients were analyzed. Remission rates and survival outcomes were evaluated. For analyses in context of ELN2022 classification, patients carrying an MR mutation were excluded from the adverse group and analyzed separately; those with co-occurring favorable or intermediate features remained in their respective groups. Overall, 1698 patients (34.1%) harbored at least one MR mutation. Lower complete remission rates were observed in MR-mutated cases (65.7% vs 77.7%; *p* < 0.001) along with shorter event-free (HR 1.45; *p* < 0.001), relapse-free (HR 1.33; *p* < 0.001), and overall survival (HR 1.45; *p* < 0.001) were recorded. Gene-specific prognostic patterns emerged: *ASXL1*, *RUNX1*, *SF3B1*, and *U2AF1* mutations associated with adverse risk-like outcomes; *SRSF2* and *STAG2* aligned with intermediate-risk; *BCOR*, *EZH2*, and *ZRSR2* did not differ significantly from intermediate or adverse risk. These findings from a large cooperative cohort highlight prognostic heterogeneity among MR mutations and suggest that SRSF2 and STAG2 mutations are associated with less adverse risk patterns, comparable to intermediate-risk.

## Introduction

Prognosis and clinical management of patients with acute myeloid leukemia (AML) is determined by the genetic profile of the underlying disease. In addition to classical cytogenetic aberrations, several molecular changes have been established as key prognostic markers. Genetic risk stratification systems such as the most widely adapted European LeukemiaNet (ELN) [1] classification categorize patients into three main groups with low, intermediate, and high risk of primary treatment failure, relapse or death. Recent updates from the ELN [1], International Consensus Classification (ICC) [2], and World Health Organization (WHO) [3] now include a novel set of molecular changes that are highly associated with secondary (s-) AML. These “myelodysplasia-related gene mutations” (MR gene mutation, also called secondary-type mutations) include mutations in the genes *SRSF2*, *SF3B1*, *U2AF1*, *ZRSR2*, *ASXL1*, *EZH2*, *BCOR*, and *STAG2*. The ICC 2022 [2] definition of MR gene mutations includes *RUNX1* mutations as an MR gene mutation while the WHO 2022 [3] only includes the aforementioned eight mutations. Several studies showed that MR gene mutations are associated with poor outcome [4–6].

Based on these findings, the updated ELN recommendations added the group of MR gene mutations (according to ICC definitions) in the absence of favorable or intermediate risk defining markers to the adverse prognostic category [1].

While several retrospective studies [7, 8] of intensively treated patients confirmed the unfavorable prognostic impact of MR gene mutations, explorative analyses of individual MR gene mutations revealed distinct differential survival outcomes amongst individual MR gene mutations, raising the question whether grouping all MR gene mutations in the adverse risk group is justified [7, 9, 10]. Because of the low prevalence of MR gene mutations in the analyzed datasets (except for *ASXL1* [11, 12] and *RUNX1* [13]), this question could not yet be answered with statistical certainty. To address this issue and analyze the prognostic impact of all individual mutations separately, genetic and clinical data from the largest group so far of intensively treated patients of five cooperative study groups from Germany, France, the Czech Republic, and Austria were collected.

## Methods

### Clinical and molecular data

For this joint analysis, genetic and clinical data of newly diagnosed and intensively treated patients with AML (excluding acute promyelocytic leukemia) were gathered and harmonized from registries and previously published clinical trials (see supplementary Table 1) of the Study Alliance Leukemia (SAL, *n* = 1608) [14–17], the AML Study Group (AMLSG, *n* = 1228) [18–20], the AML Cooperative Group (AMLCG, *n* = 1137) [21, 22], the French DATAML registry (*n* = 831) [23], and the Czech Leukemia Study Group for Life (CELL, *n* = 174). Patients were enrolled between 1998 and 2021.

Clinical data were provided from each study group. In general, pre-treatment samples from bone marrow or peripheral blood were used for screening for cytogenetic aberrations and molecular alterations in all patients by each study group. Standard techniques for chromosome banding, fluorescence-in-situ-hybridization and molecular analysis were used as previously described [15–17]. Patients were assigned to risk groups according to recommendations of the ELN 2022 guidelines [1].

### Ethics approval and consent to participate

Written informed consent was obtained from all patients in accordance with the revised Declaration of Helsinki [24]. All studies were approved by the local Institutional Review Board (Technical University Dresden [EK 98032010]).

### Statistical analysis

Normality of data was assessed using the Shapiro-Wilk test. If the assumption of normality was met, continuous variables between two groups were compared using the two-sided unpaired *t* test. If the assumption of normality was not met, continuous variables between two groups were analyzed using the Wilcoxon rank sum test. Fisher’s exact test was used to compare categorical variables. Standard clinical endpoints were determined according to ELN 2022 recommendations [1]. The odds ratio (OR) for complete remission (CR) after intensive induction therapy was evaluated using logistic regression models. Time-to-event variables including event-free survival (EFS), relapse-free survival (RFS), and overall survival (OS) were analyzed using Cox proportional hazard models to obtain hazard ratios (HR) as well as the Kaplan-Meier method and the log-rank test. For all OR and HR, 95%-confidence intervals (95%-CI) are reported. All tests were carried out as two-sided tests. Statistical significance was determined using a significance level α of 0.05. All analyses were performed, and visualizations were created in STATA BE 18.0 (Stata Corp, College Station, TX, USA) and Python 3.11 (Python Software Foundation, Wilmington, DE, USA).

## Results

### Characteristics of acute myeloid leukemia patients according to the myelodysplasia-related gene mutation status

**Fig. 1:**
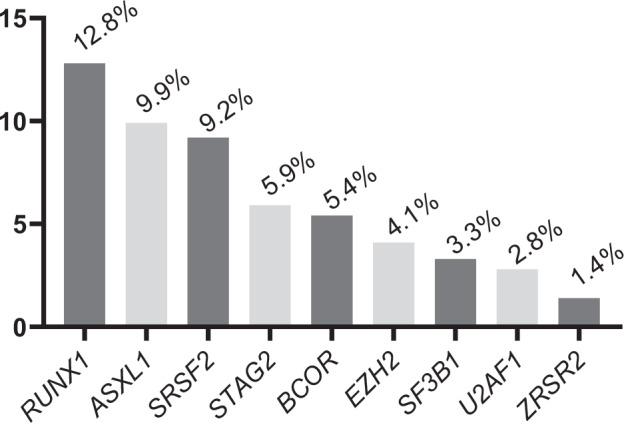
Prevalence of Myelodysplasia-Related Gene Mutations. Prevalence of myelodysplasia-related gene mutations in a cohort of 4978 intensively treated patients with acute myeloid leukemia.

In the entire cohort of 4978 intensively treated AML patients, 1698 patients were found to carry MR gene mutations according to the ICC 2022 definition (Table 1). *RUNX1* mutations had the highest prevalence, followed by mutations in *ASXL1*, *SRSF2*, *STAG2*, *BCOR*, *EZH2*, *SF3B1*, *U2AF1*, and *ZRSR2* (Fig. 1).

**Table 1:** Baseline patient characteristics with respect to MR gene mutation status.

Variable	MR gene mutation	No MR gene mutation	*p*
**n/N (%)**	1698/4978 (34.1)	3280/4978 (65.9)	
**Age (years), median (IQR)**	59 (50–67)	52 (42–61)	**<0.001**
**Sex, n (%)**			**<0.001**
female	660 (38.9)	1703 (51.9)	
male	1038 (61.1)	1577 (48.1)	
**Disease status, n (%)**			
de novo	1287 (75.8)	2896 (88.3)	**<0.001**
sAML	326 (19.2)	208 (6.3)	**<0.001**
tAML	71 (4.2)	151 (4.6)	0.515
missing	14 (0.8)	25 (0.8)	
**Complex karyotype, n (%)**			**<0.001**
Yes	131 (7.7)	383 (11.7)	
No	1513 (89.1)	2799 (85.3)	
missing	54 (3.2)	98 (3.0)	
**Normal karyotype, n (%)**			0.285
Yes	893 (52.6)	1781 (54.3)	
No	751 (44.2)	1401 (42.7)	
missing	54 (3.2)	98 (3.0)	
**Allo HSCT in CR1, n (%)**			0.737
Yes	335 (19.7)	660 (20.1)	
No	1361 (80.2)	2613 (79.7)	
missing	2 (0.1)	7 (0.2)	
**Allo HSCT as salvage therapy, n (%)**			**0.030**
Yes	258 (15.2)	605 (18.4)	
No	1232 (72.6)	2416 (73.7)	
missing	208 (12.2)	259 (7.9)	
**Laboratory, median (IQR)**			
WBC (10^9^/l)	10.7 (2.9–39.6)	23.3 (5.8–66.0)	**<0.001**
Hb (mmol/l)	5.7 (4.9–6.6)	5.8 (5.0–6.7)	0.160
PLT (10^9^/l)	55 (30–105)	55 (30–100)	0.570
PB blasts (%)	26 (6–63)	43 (13–76)	**<0.001**
BM blasts (%)	60 (39–80)	72 (50–88)	**<0.001**

Baseline patient characteristics are shown. Boldface indicates statistical significance (*p* < 0.05).

*AML* acute myeloid leukemia, *sAML* secondary AML, *tAML* therapy-associated AML, *allo* allogeneic, *BM* bone marrow, *HB* hemoglobin, *HSCT* hematopoietic stem cell transplantation, *IQR* interquartile range, *n/N* number, *PB* peripheral blood, *PLT* platelet count, *WBC* white blood cell count, *wt* wildtype.

MR gene mutation patients were significantly older than non-MR gene mutation patients. MR gene mutations were significantly associated with male sex and s-AML. The proportion of therapy-associated AML (t-AML) did not differ between MR gene mutation and non-MR gene mutation patients. While the prevalence of normal karyotype did not differ between MR gene mutation and non-MR gene mutation patients, complex karyotypes were significantly less prevalent in MR gene mutation patients. Patients with MR gene mutations presented with a significantly lower white blood cell (WBC) count as well as significantly lower bone marrow and peripheral blood blast counts at initial diagnosis, while platelet count and hemoglobin levels did not differ between mutated and unmutated patients. Similar results were found for the WHO 2022 definition of MR gene mutations (see Supplemental Material).

### Patients’ characteristics according to the individual myelodysplasia-related gene mutations

Patients with mutations in *ASXL1*, *SRSF2*, and *U2AF1* had the highest median age at diagnosis with 63 years in each subgroup. Gene mutations in *ASXL1*, *SRSF2*, *STAG2*, *U2AF1*, *ZRSR2*, and *RUNX1* were significantly associated with male sex. Except for *ZRSR2* alterations, all individual MR gene mutations were associated with significantly lower rates of de novo AML and, conversely, with significantly higher rates of s-AML. For t-AML, frequencies did not differ between any individual MR gene mutation and their respective wildtypes.

The prevalence of complex karyotype was significantly lower in AML with alterations of *ASXL1*, *EZH2*, *SRSF2*, or *STAG2*. Significantly lower white blood cell counts at initial diagnosis were found for AML patients with alterations in *ASXL1*, *BCOR*, *EZH2*, *SRSF2*, *STAG2*, *U2AF1*, and *RUNX1*. Significantly lower peripheral and bone marrow blast counts were found for AML patients with mutations in *ASXL1*, *SF3B1*, *SRSF2*, *STAG2*, *U2AF1*, and *RUNX1*, while for *BCOR-* and *EZH2*-mutated AML, only peripheral blood blast counts were significantly lower. Platelet counts were significantly higher for *SF3B1* -mutated AML. For *ZRSR2* -mutated AML, no significant differences were observed regarding any analyzed pretreatment marker. Baseline characteristics for individual MR gene mutations are summarized in Supplementary Tables S2–S12.

### Outcome analyses according to myelodysplasia-related gene mutation status

After intensive induction therapy, 65.7% of patients with MR gene mutations achieved a CR compared to 77.7% of patients without MR gene mutations (OR 0.55, *p* < 0.001). In the entire cohort, median EFS was significantly shorter for patients with MR gene mutations compared to non-MR gene mutation patients (HR: 1.45, *p* < 0.001, Table 2). The same pattern was observed for RFS (HR: 1.33, *p* < 0.001; Table 2) and OS (HR: 1.45, *p* < 0.001; Table 2).

**Table 2:** Summary of patient outcome with respect to myelodysplasia-related gene mutations (MR gene mutation) status.

Outcome	MR gene mutation	No MR gene mutation	OR/HR	*p*
n/N (%)	1698/4978 (34.1)	3280/4978 (65.9)		
CR rate, n (%)	1116/1698 (65.7)	2549/3280 (77.7)	0.55 [0.58–0.63]	**<0.001**
EFS	6.3 [5.4–7.0]	10.5 [9.8–11.3]	1.45 [1.36–1.55]	**<0.001**
RFS	14.3 [12.7–16.3]	20.3 [17.9–23.3]	1.33 [1.22–1.45]	**<0.001**
OS	16.6 [15.2–17.8]	26.5 [24.3–30.2]	1.45 [1.35–1.56]	**<0.001**

Survival times are displayed in months with the median value and the 95%-confidence interval in square brackets. Boldface indicates statistical significance (*p* < 0.05).

*CR* complete remission, *EFS* event-free survival, *HR* hazard ratio, *neg.* negative, *n/N* number, *OR* odds ratio, *OS* overall survival, *pos.* positive, *RFS* relapse-free-survival.

Outcomes were also analyzed in the subgroup of patients who underwent allogeneic stem cell transplantation in first complete remission (Supplementary Table S13). In this cohort, the differences in outcomes that were observed between patients with and without MR gene mutations in the overall population were no longer evident for EFS and RFS. However, patients with an MR gene mutation had a significantly shorter OS than patients without an MR gene mutation after transplantation.

The prognostic impact of the individual MR gene mutations is shown in the Supplementary Tables S14–S22.

### Prognostic impact of grouped myelodysplasia-related gene mutations in relation to ELN 2022

To relate the prognostic impact of grouped MR gene mutations to the established ELN 2022 risk categories, patients with MR gene mutations and co-occurring genetic changes that define ELN 2022 risk groups were classified into their respective ELN 2022 categories regardless of the MR gene mutation status. Specifically, patients with co-occurring favorable risk features (i.e., core-binding factor AML [*n* = 44, 0.8%], mutated *NPM1* without *FLT3*-ITD [*n* = 163, 3.0%], or bZIP in-frame mutated *CEBPA* [*n* = 23, 0.4%]) were assigned to the ELN 2022 favorable risk group regardless of the MR gene mutation status. Similarly, those with intermediate risk features that take precedence (*i.e*., t(9;11)(p21.3;q23.3) [*n* = 14, 0.3%]) were categorized under the ELN 2022 intermediate risk group. All other patients with MR gene mutation and no other defining ELN 2022 adverse risk features (i.e., no t(6;9), t(v;11q23.3), t(9;22), t(8;16), inv(3) or t(3;3), t(3q26.2;v), −5 or del(5q), −7, −17/abn(17p), or complex karyotype), were separated from non-MR gene mutation ELN 2022 adverse risk patients and analyzed in relation to the ELN 2022 risk groups.

**Fig. 2:**
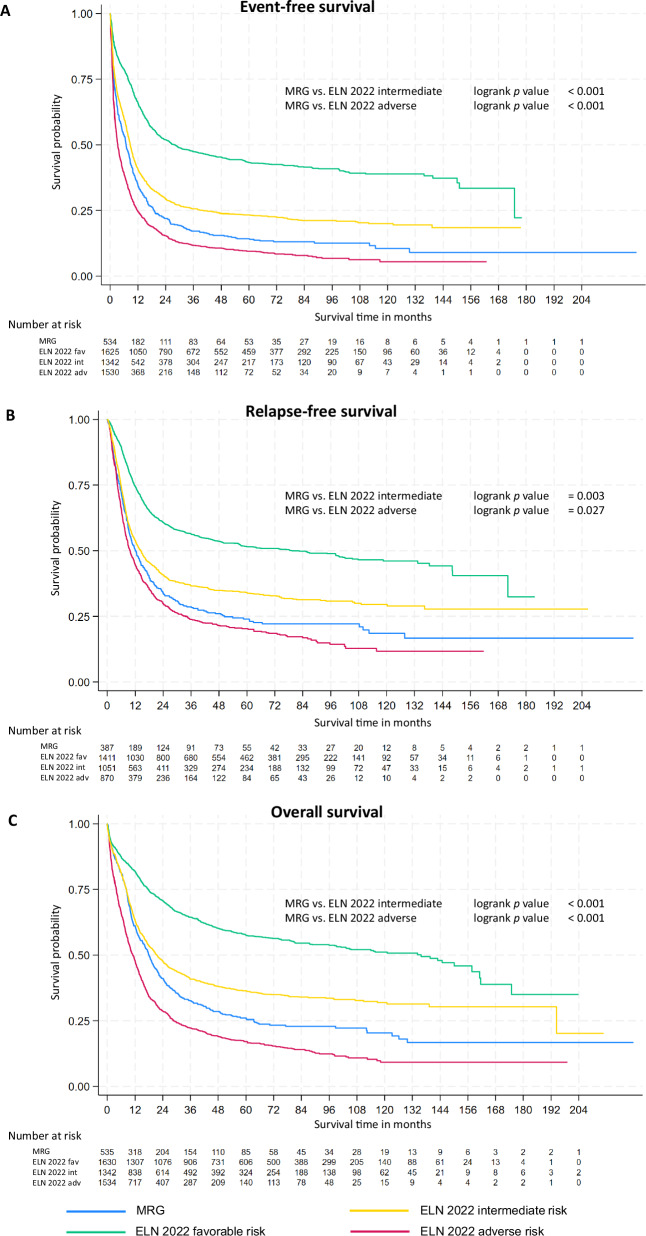
Kaplan-Meier plots comparing ELN 2022 risk groups to AML with myelodysplasia-related gene mutations. Patients from the entire cohort were retrospectively assigned to ELN 2022 risk groups. Patients within the ELN 2022 adverse risk group that had MR gene mutation were treated as a separate group for this Kaplan-Meier analysis: Event-free survival (EFS, panel **A**), relapse-free survival (RFS, panel **B**), and overall survival (OS, panel **C**). Log rank *p* values are reported for the distinction between patients with MR gene mutation and patients with ELN 2022 adverse risk (MR gene mutation excluded).

The median EFS for MR gene mutation patients was 4.9 months compared to 2.7 months for ELN 2022 adverse risk patients without MR gene mutations (*p* < 0.001; Table 3; Fig. 2 A). In contrast, median EFS for the ELN 2022 favorable group was 28.1 months, and 8.6 months for ELN 2022 intermediate. Likewise, median RFS was longer for patients with MR gene mutations (11.9 months) compared to ELN 2022 adverse risk patients without MR gene mutations (7.4 months, *p* < 0.001; Fig. 2B). Lastly, the same pattern was observed for OS: MR gene mutation patients without favorable or intermediate genetics had a longer median OS (14.7 months) than non-MR gene mutation ELN 2022 adverse risk patients with a median OS of 8.3 months (*p* < 0.001, Fig. 2C).

**Table 3:** Summary of outcomes of MR gene mutation patients compared to ELN 2022 risk groups.

	ELN 2022 favorable	ELN 2022 intermediate	ELN 2022 adverse*	MR gene mutation
**EFS**				
median	28.1 [22.5–35.8]	8.6 [7.9–9.4]	3.2 [2.9–3.7]	6.8 [5.6–8.1]
HR	0.42 [0.39–0.46]	1.04 [0.97-1.12]	2.12 [1.98–2.27]	1.34 [1.21–1.47]
*p*	**<0.001**	0.238	**<0.001**	**<0.001**
**RFS**				
median	79.4 [56.4–105.3]	14.4 [12.3–16.4]	9.8 [8.7–11.2]	11.9 [10.1–14.4]
HR	0.48 [0.44–0.52]	1.15 [1.05–1.26]	1.84 [1.68–2.01]	1.42 [1.26–1.61]
*p*	**<0.001**	**0.002**	**<0.001**	**<0.001**
**OS**				
median	135.9 [103.2–157.7]	21.5 [18.9–24.3]	11.0 [10.0–12.1]	17.9 [15.7–20.1]
HR	0.40 [0.37–0.44]	0.99 [0.91–1.07]	2.2 [2.09–2.42]	1.27 [1.14–1.41]
*p*	**<0.001**	0.737	**<0.001**	**<0.001**

Survival is reported for the ICC definition of MR gene mutations. Survival times are displayed in months. Square brackets show 95%-confidence intervals. Boldface indicates statistical significance (*p* < 0.05). All patients in the cohort were retrospectively assigned to ELN 2022 risk groups. For this analysis, patients with MR gene mutation were only considered for the MR gene mutation group if no other class-defining alterations were present. For instance, if a patient was originally only assigned to ELN 2022 adverse risk because of the presence of an MR gene mutation, this patient was allocated to the respective MR gene mutation group. Vice versa, if MR gene mutation bearing patients had co-occurring markers of favorable, intermediate (t(9;11)), or adverse risk, they were allocated to the respective ELN group. Hence, patients in the MR gene mutation group have no other risk-defining markers except for MR gene mutation 3.

### Prognostic impact of individual myelodysplasia-related gene mutations in relation to ELN 2022

In accordance with the analyses described above, individual MR gene mutations were separated from the ELN 2022 adverse risk group and plotted separately while patients harboring an individual MR gene mutation with co-occurring features that define a favorable or intermediate risk remained in the respective risk group (Fig. 3 and Supplementary Figs. S1–S9).

**Fig. 3:**
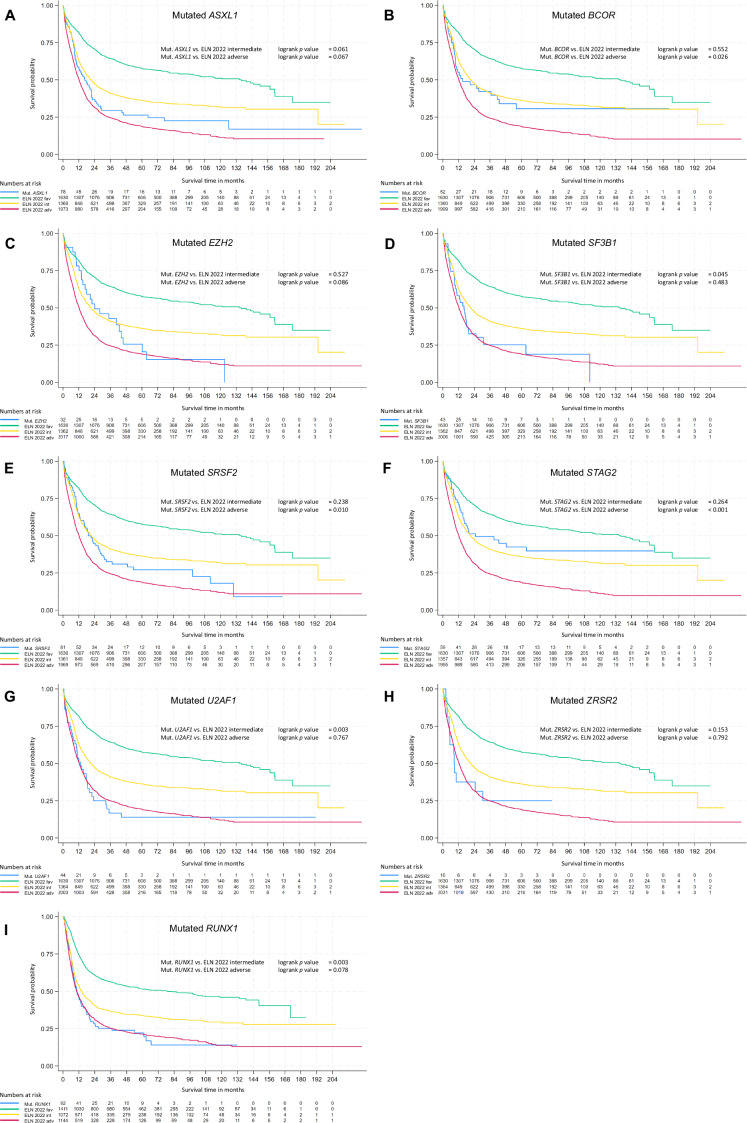
Kaplan-Meier plots for overall survival comparing ELN 2022 risk groups to the individual myelodysplasia-related gene mutations. Patients from the entire cohort were retrospectively assigned to ELN 2022 risk groups. Patients within the ELN 2022 adverse risk group that had MR gene mutation were treated as a separate group for this Kaplan-Meier analysis. The individual gene mutations are (**A**) *ASXL1*, (**B**) *BCOR*, (**C**) *EZH2*, (**D**) *SF3B1*, (**E**) *SRSF2*, (**F**) *STAG2*, (**G**) *U2AF1* and (**H**) *ZRSR2*. Log rank *p* values are reported for the distinction between patients with MR gene mutation and patients with ELN 2022 adverse risk (MR gene mutation excluded).

Patients with *ASXL1*, *RUNX1*, *SF3B1*, or *U2AF1* mutations had significantly shorter median EFS than patients in the ELN intermediate risk group, with no significant differences compared to the adverse risk group (Table 4). Conversely, patients with alterations in *SRSF2* or *STAG2* showed no survival difference compared to ELN 2022 intermediate risk patients but a significantly longer EFS than adverse risk patients. For *BCOR*, *EZH2*, and *ZRSR2* mutations, EFS was in between the intermediate or adverse risk groups with no statistically significant differences compared to either risk group.

**Table 4:** Summary of outcomes of individual MR gene mutation patients compared to ELN 2022 risk groups.

	Event-free survival		Relapse-free survival		Overall survival	
	ELN 2022 intermediate	ELN 2022 adverse	ELN 2022 intermediate	ELN 2022 adverse	ELN 2022 intermediate	ELN 2022 adverse
***ASXL1***	0.019^#^	n.s.^#^	n.s.	n.s.	n.s.	n.s.
***BCOR***	n.s.	n.s.	0.011^#^	n.s.^#^	n.s.^§^	0.026^§^
***EZH2***	n.s.	n.s.	n.s.	n.s.	n.s.	n.s.
***RUNX1***	<0.001^#^	n.s.^#^	0.014^#^	n.s.^#^	0.003^#^	n.s.^#^
***SF3B1***	0.003^#^	n.s.^#^	0.046^#^	n.s.^#^	0.045^#^	n.s.^#^
***SRSF2***	n.s.^§^	<0.001^§^	n.s.	n.s.	n.s.^§^	0.01^§^
***STAG2***	n.s.^§^	<0.001^§^	n.s.^§^	<0.001^§^	n.s.^§^	<0.001^§^
***U2AF1***	0.003^#^	n.s.^#^	n.s.	n.s.	0.003^#^	n.s.^#^
***ZRSR2***	n.s.	n.s.	n.s.	n.s.	n.s.	n.s.

Summarized are the *p*-values for statistically significant findings from the log-rank test shown in Supplementary Figs. S1–S9. A *#* indicates that these mutations are associated with a significantly shorter outcome compared to the ELN 2022 intermediate-risk group, but there is no significant difference compared to the adverse-risk group. A *§* signifies no significant difference from the intermediate-risk group, but a significantly better outcome compared to the adverse-risk group. All patients in the cohort were retrospectively assigned to ELN 2022 risk groups. For this analysis, patients with MR gene mutation were only considered for the MR gene mutation group if no other class-defining alterations were present. For instance, if a patient was originally only assigned to ELN 2022 adverse risk because of the presence of an MR gene mutation, this patient was allocated to the respective MR gene mutation group. Vice versa, if MR gene mutation bearing patients had co-occurring markers of favorable, intermediate (t(9;11)), or adverse risk, they were allocated to the respective ELN group. Hence, patients in the MR gene mutation group have no other risk-defining markers except for MR gene mutation.

For RFS, mutations in *BCOR*, *RUNX1*, and *SF3B1* again were associated with significantly shorter survival compared to patients in the intermediate risk group but no significant difference to the adverse risk group. In contrast, RFS for patients with *STAG2* mutations showed no difference from the intermediate risk group and was significantly longer than for patients in the ELN 2022 adverse risk group. Patients with an *ASXL1*, *EZH2*, *SRSF2*, *U2AF1*, or *ZRSR2* mutation had no significant different RFS compared to intermediate and adverse risk patients.

Regarding OS, mutations in *RUNX1*, *SF3B1*, and *U2AF1* were associated with significantly shorter OS compared to patients in the ELN intermediate risk group, with no significant differences observed relative to the adverse risk group. On the other hand, mutations in *BCOR*, *SRSF2*, and *STAG2* showed a significantly longer OS than adverse risk patients but no difference to intermediate risk patients. Mutations in *ASXL1*, *EZH2*, or *ZRSR2* showed no significant differences either to intermediate or adverse risk groups.

Results of significance tests for survival differences between individual MR gene mutations and ELN 2022 risk groups are displayed in Table 4.

## Discussion

ELN as well as the ICC and WHO emphasized the significance of MR gene mutations in their latest updates based on the distinct AML biology and clinical outcomes. The large number of patients with MR gene mutations in the present cohort allowed us to reassess characteristics and prognostic patterns of grouped MR gene mutations in more detail and with higher statistical certainty. Most importantly, the sample size allowed us for the first time to separate the prognostic impact of individual mutations in the context of the ELN 2022 classification.

In accordance with Lindsley et al. [4], all MR gene mutations, except for mutated *ZRSR2*, were more frequently observed in s-AML in the whole cohort, underlining their significance in the pathogenesis of s-AML. As expected and consistent with other studies [6, 25–29], patients with MR gene mutations were older, showed a lower WBC count, a lower percentage of BM blasts at diagnosis and were more likely to be male compared to those without MR gene mutations.

Several studies [4–6] showed that the presence of an MR gene mutation is associated with poorer outcomes. However, these studies combined all MR gene mutations, so the prognostic impact of individual MR gene mutations remained unclear, partly due to variations in their prevalence. In the analyzed cohort, most frequent MR gene mutations were *RUNX1* mutations with 12%, while *ZRSR2* mutations were the least common, found in only 1.4% of AML patients. This disparity in frequency explains why the prognostic impact of the more common MR gene mutations - such as in *RUNX1* [13], *ASXL1* [11, 12], *SRSF2* [6, 30], and *EZH2* [31] - has been previously studied and unanimously linked to worse outcomes. In this cohort, the presence of MR gene mutations—treated as a combined “one-for-all” variable according to both ICC and WHO definitions—as well as most individual mutations, was associated with worse outcomes.

To provide greater clinical relevance to the findings, the data were analyzed within the context of the ELN 2022 classification [1], which is the most widely used risk stratification system for AML to guide therapeutic decision-making in patients eligible for intensive chemotherapy. Consistent with previous studies [7, 8], MR gene mutation patients demonstrated worse outcomes compared to those classified as ELN intermediate or favorable risk. However, it was also observed that MR gene mutation patients without co-occurring adverse risk markers had better outcomes than patients in the adverse risk category. Similar to findings reported by Mrózek et al. [7], the present analysis suggests that the isolated presence of MR gene mutations is associated with an outcome falling between intermediate and adverse risk groups.

Looking at the different mutations, three distinct categories were identified: First, the MR gene mutations in *ASXL1*, *RUNX1*, *SRSF2*, and *U2AF1* were associated with outcomes significantly worse than those of intermediate risk patients but similar to adverse risk patients for at least one clinical endpoint. Notably, the presence of mutations in *ASXL1* and *RUNX1* was already classified as adverse risk in the ELN2017 classification, and prior studies have shown that their outcomes are comparable to those of other adverse-risk patients [27, 32]. Second, mutations in *EZH2* and *ZRSR2* were associated with outcomes neither significantly worse than those of intermediate risk patients nor significantly better than those of adverse risk patients. Third, mutations in *SRSF2* and *STAG2* were found to be linked to significantly better outcomes, comparable to those classified as intermediate risk rather than adverse risk. Consequently, the data suggest that mutations in *SRSF2* and *STAG2* should probably not be classified as adverse.

An exception from these three categories are mutations in *BCOR*, as these are associated with a significantly shorter RFS compared to intermediate risk patients but a significantly better OS than adverse risk patients. This suggests that *BCOR*-mutated patients respond well to salvage treatment in relapse, although further analyses are required to confirm this hypothesis.

This study represents the largest published analysis of individual MR gene mutations to date. Limitations include the retrospective nature of the analysis with drawbacks such as selection, heterogeneity in cytostatic treatment, and supportive care. Although most patients in this cohort received a 7 + 3-based induction regimen, novel therapies, such as gemtuzumab ozogamicin, FLT3 inhibitors, and CPX-351, were not necessarily available or standard of care during the period of data collection.

Based on a large international retrospective data set, this study showed that the majority of MR gene mutations are associated with dismal outcomes, while mutations in *SRSF2* and *STAG2* show a better prognosis, aligning them with the ELN intermediate rather than adverse risk category.

## Supplementary information

Supplemental Material

## Data Availability

Data is available upon request to the corresponding author.
