# Supplementary material for: Differential prognostic impact of myelodysplasia-related gene mutations in a European cohort of 4978 intensively treated AML patients
Source: Leukemia. 2025 Oct 27;40(1):63–71. doi: 10.1038/s41375-025-02781-6 (PMC12789037; doi:10.1038/s41375-025-02781-6)
Supplement: Supplementary file 1 — Supplemental Material [file 41375_2025_2781_MOESM1_ESM.docx]

**Supplemental Material for**

**“Differential Prognostic Impact of Myelodysplasia-Related Gene Mutations in a European Cohort of 4,978 Intensively Treated AML Patients”**

### **Characteristics of Acute Myeloid Leukemia Patients According to the WHO 2022 Myelodysplasia-Related Gene Status**

In a cohort of 4,978 intensively treated AML patients, we identified 1,485 patients meeting the WHO 2022 definition for MR gene mutations. In our cohort, *ASXL1* mutations had the highest prevalence, followed by *SRSF2*, *STAG2*, *BCOR*, *EZH2*, *SF3B1*, *U2AF1*, and *ZRSR2*.

Patients with MR gene mutations, as defined by WHO 2022, were significantly older than those without MR mutations. These mutations were also significantly associated with male sex and secondary AML. While the prevalence of a normal karyotype did not differ between patients with and without MR gene mutations, complex karyotypes were significantly less common in MR gene mutated patients.

Additionally, patients with MR gene mutations had significantly lower white blood cell (WBC) counts, as well as lower bone marrow and peripheral blood blast counts at diagnosis. However, platelet count and hemoglobin levels did not differ between mutated and unmutated patients (Supplement Table S2).

**Prognostic Impact of Grouped Myelodysplasia-Related Gene Mutations according to the WHO 2022 definition in Relation to ELN 2022**

The median event-free survival (EFS) for patients with MR gene mutations was 4.7 months, compared to 3.0 months for ELN 2022 adverse risk patients without MR gene mutations (p<0.001; Supplement Table S3). In contrast, the median EFS was 28.1 months for the ELN 2022 favorable risk group and 8.6 months for the intermediate risk group. Similarly, median relapse-free survival (RFS) was longer in patients with MR gene mutations (12.6 months) than in ELN 2022 adverse risk patients without MR gene mutations (8.0 months, p<0.001; Supplement Table S3). A similar result was observed for overall survival (OS), where patients with MR gene mutations but without favorable or intermediate risk features had a longer median OS (14.6 months) compared to ELN 2022 adverse risk patients without MR gene mutations, who had a median OS of approximately 9.5 months (p < 0.001).

| **study group** | **trial name** | **clinicaltrials.gov identifier** | **trial duration** | **protocol summary** |
| --- | --- | --- | --- | --- |
| SAL | AML96 | NCT00180115 | 1996-2008 | risk-adapted postremission treatment regarding allogeneic stem cell transplantation for high-risk AML and related allogeneic and autologous stem cell transplantation for standard-risk AML, and randomization between intermediate-dose and high-dose cytarabine within the first post-remission course |
| SAL | AML2003 | NCT00180102 | 2003-2009 | early allogeneic stem cell transplantation in post-induction aplasia for high-risk AML, factorial design with four therapy arms with two factors of two stages (intensified vs. standard therapy and cytarabine vs. cytarabine + mitoxantrone + amsacrin) |
| SAL | AML60+ | NCT00180167 | 2005-2010 | Patients ≥ 60 years, mitoxantron on day 1,2,3 + cytarabine on days 1,3,5,7 vs. DA 7+3 |
| SAL | SORAML | NCT00893373 | 2011-2014 | Standard therapy + sorafenib vs. standard therapy + placebo |
| SAL | SAL registry | NCT03188874 | 2010-present | Prospective registry of AML patients |
| AMLCG | AMLCG-1999 | NCT00266136 | 1999-2007 | double induction with HAM-HAM, multiple course G-CSF or myeloablative consolidation with Bu/Cy and autologous blood stem cell transplantation instead of maintenance vs. standard therapy |
| AMLCG | AMLCG-2008 | NCT01382147 | 2008-2012 | S-HAM escalated for younger patients and S-HAM basis for elderly patients vs. TAD-HAM (younger) or HAM-HAM (elderly) |
| AMLSG | AMLSG0704 | NCT00151242 | 2004-2011 | Two cycles of intensive induction chemotherapy with or without open-label ATRA |
| AMLSG | AMLHD98A | NCT00146120 | 1998-2004 | Risk-adapted (cytogenetics and treatment response-related) treatment comparing matched related and matched unrelated donors for allogeneic transplantation |
| DATAML | N/A | N/A | 2000-present | Prospective registry of AML patients treated by intensive chemotherapy according to FILO protocols |
| CELL | DATOOL-AML-registry | N/A | 2014-present | Prospective registry of AML patients |

**Table S1 Overview of source clinical trials**.

| **Variable** | **MR gene mutation** | **no MR gene mutation** | ***p*** |
| --- | --- | --- | --- |
| **n/N (%)** | 1485/4978 (29.8) | 3493/4978 (70.1) |  |
| **Age (years), median (IQR)** | 60 (51-58) | 53 (42-61) | **<0.001** |
| **Sex, n (%)** |  |  | **<0.001** |
| female | 557 (37.5) | 1806 (51.7) |  |
| male | 928 (62.5) | 1687 (48.3) |  |
| **Disease status,  n (%)** |  |  |  |
| de novo | 1121 (75.5) | 3062 (87.7) | **<0.001** |
| sAML | 294 (19.8) | 240 (6.9) | **<0.001** |
| tAML | 57 (3.8) | 165 (4.7) | 0.177 |
| missing | 13 (0.9) | 26 (0.7) |  |
| **Complex karyotype,  n (%)** |  |  | **<0.001** |
| Yes | 98 (6.6) | 416 (11.9) |  |
| No | 1342 (90.4) | 2970 (85.0) |  |
| missing | 45 (3.0) | 107 (3.1) |  |
| **Normal karyotype,  n (%)** |  |  | 0.635 |
| Yes | 790 (53.2) | 1884 (53.9) |  |
| No | 650 (43.8) | 1502 (43.0) |  |
| missing | 45 (3.0) | 107 (3.1) |  |
| **Allo HSCT in CR1, n (%)** |  |  | 0.394 |
| Yes | 286 (19.3) | 709 (20.3) |  |
| No | 1198 (80.7) | 2776 (79.5) |  |
| missing | 1 (<0.0) | 8 (0.2) |  |
| **Allo HSCT as salvage therapy, n (%)** |  |  | **0.003** |
| Yes | 213 (14.3) | 650 (18.6) |  |
| No | 1085 (73.1) | 2563 (73.4) |  |
| missing | 187 (12.6) | 280 (8.0) |  |
| **Laboratory, median (IQR)** |  |  |  |
| WBC (10^9^/l) | 10.4 (2.8-39.0) | 22.5 (5.6-65.0) | **<0.001** |
| Hb (mmol/l) | 5.8 (5.0-6.6) | 5.7 (5.0-6.6) | 0.941 |
| PLT (10^9^/l) | 53 (30-101) | 56 (30-101) | 0.647 |
| PB blasts (%) | 24 (5-61) | 42 (13-76) | **<0.001** |
| BM blasts (%) | 60 (37-80) | 72 (50-87) | **<0.001** |

**Table S2 Baseline patient characteristics with respect to MR gene mutation status.** The novel International Consensus Classification 2022 (ICC) and World Health Organization 2022 (WHO) definitions of MR gene mutation vary by the inclusion or exclusion of *RUNX1* mutations. Baseline patient characteristics are shown for the WHO 2022 classification systems. Abbreviations: acute myeloid leukemia (AML), secondary AML (sAML), therapy-associated AML (tAML), allogeneic (allo), bone marrow (BM), hemoglobin (HB), hematopoietic stem cell transplantation (HSCT), interquartile range (IQR), number (n/N), peripheral blood (PB), platelet count (PLT), white blood cell count (WBC), wildtype (wt). Boldface indicates statistical significance (*p*<0.05).

|  | **ELN 2022**  **favorable** | **ELN 2022**  **intermediate** | **ELN 2022**  **adverse*** | **MR gene mutation^**^** |
| --- | --- | --- | --- | --- |
| **EFS** |  |  |  |  |
| median | 28.1 [22.5-35.8] | 8.6 [7.9-9.4] | 3.3 [3.0-3.8] | 6.9 [5.6-8.5] |
| HR | 0.42 [0.39-0.46] | 1.04 [0.97-1.12] | 2.14 [2.01-2.29] | 1.28 [1.14-1.43] |
| *p* | **<0.001** | 0.238 | **<0.001** | **<0.001** |
| **RFS** |  |  |  |  |
| median | 79.4 [56.4-105.3] | 14.4 [12.3-16.4] | 9.8 [8.8-11.2] | 12.6 [10.0-16.1] |
| HR | 0.48 [0.44-0.52] | 1.15 [1.05-1.26] | 1.87 [1.71-2.04] | 1.36 [1.18-1.56] |
| *p* | **<0.001** | **0.002** | **<0.001** | **<0.001** |
| **OS** |  |  |  |  |
| median | 135.9 [103.2-157.7] | 21.5 [18.9-24.3] | 11.5 [10.3-12.3] | 17.8 [15.3-20.1] |
| HR | 0.40 [0.37-0.44] | 0.99 [0.91-1.07] | 2.26 [2.10-2.42] | 1.21 [1.08-1.37] |
| *p* | **<0.001** | 0.737 | **<0.001** | **0.002** |

**Supplemental Table S3 Summary of outcomes of MR gene mutation patients compared to ELN 2022 risk groups.** Survival is reported for WHO definitions of MR gene mutation. Survival times are displayed in months. Square brackets show 95%-confidence intervals. Boldface indicates statistical significance (p<0.05). All patients in the cohort were retrospectively assigned to ELN 2022 risk groups. *For this analysis, patients with MR gene mutation were only considered for the MR gene mutation group if no other class-defining alterations were present. For instance, if a patient was originally only assigned to ELN 2022 adverse risk because of the presence of an MR gene mutation, this patient was allocated to the respective MR gene mutation group. Vice versa, if MR gene mutation bearing patients had co-occurring markers of favorable, intermediate, or adverse risk, they were allocated to the respective ELN group. Hence, patients in the MR gene mutation group have no other risk-defining markers except for MR gene mutation.

| **Variable** | ***ASXL1* mutated** | ***ASXL1* wildtype** | ***p*** |
| --- | --- | --- | --- |
| **n/N (%)** | 494/5311 (9.3) | 4692/5311 (88.3) |  |
| missing | 125/5311 (2.4) | |  |
| **Age (years), median (IQR)** | 63 (55-69) | 54 (44-63) | **<0.001** |
| **Sex, n (%)** |  |  | **<0.001** |
| female | 159 (32.2) | 2302 (49.1) |  |
| male | 335 (67.8) | 2390 (50.9) |  |
| **Disease status, n (%)** |  |  |  |
| de novo | 335 (67.8) | 4020 (85.7) | **<0.001** |
| sAML | 137 (27.7) | 416 (8.9) | **<0.001** |
| tAML | 16 (3.2) | 223 (4.8) | 0.142 |
| missing | 6 (1.2) | 33 (0.7) |  |
| **Complex karyotype, n (%)** |  |  | **<0.001** |
| Yes | 26 (5.3) | 522 (11.1) |  |
| No | 454 (91.9) | 4030 (85.9) |  |
| missing | 14 (2.8) | 140 (3.0) |  |
| **Normal karyotype, n (%)** |  |  | **0.043** |
| Yes | 243 (49.2) | 2525 (53.8) |  |
| No | 237 (48.0) | 2027 (43.2) |  |
| missing | 14 (2.8) | 140 (3.0) |  |
| **Laboratory, median (IQR)** |  |  |  |
| WBC (10^9^/l) | 10.8 (2.8-42.2) | 18.6 (4.5-57.8) | **<0.001** |
| HB (mmol/l) | 5.7 (5.1-6.5) | 5.8 (5.0-6.6) | 0.933 |
| PLT (10^9^/l) | 55 (32-108) | 55 (30-102) | 0.521 |
| PBB (%) | 20 (4-51) | 39 (11-74) | **<0.001** |
| BMB (%) | 51 (31-75) | 70 (47-86) | **<0.001** |

**Table S4 Baseline patient characteristics with respect to *ASXL1* mutation status.**Abbreviations: acute myeloid leukemia (AML), secondary AML (sAML), therapy-associated AML (tAML), bone marrow blasts (BMB), hemoglobin (HB), interquartile range (IQR), number (n/N), peripheral blood blasts (PBB), platelet count (PLT), white blood cell count (WBC). Boldface indicates statistical significance (*p*<0.05).

| **Variable** | ***BCOR* mutated** | ***BCOR* wildtype** | ***p*** |
| --- | --- | --- | --- |
| **n/N (%)** | 269/5311 (5.0) | 4917/5311 (92.6) |  |
| missing | 125/5311 (2.4) | |  |
| **Age (years), median (IQR)** | 60 (49-67) | 55 (44-64) | **<0.001** |
| **Sex, n (%)** |  |  | 0.531 |
| female | 133 (49.4) | 2328 (47.3) |  |
| male | 136 (50.6) | 2589 (52.7) |  |
| **Disease status, n (%)** |  |  |  |
| de novo | 208 (77.3) | 4147 (84.3) | **0.004** |
| sAML | 48 (17.8) | 505 (10.3) | **<0.001** |
| tAML | 10 (3.7) | 229 (4.7) | 0.652 |
| missing | 3 (1.1) | 36 (0.7) |  |
| **Complex karyotype, n (%)** |  |  | 0.065 |
| Yes | 19 (7.1) | 529 (10.8) |  |
| No | 241 (89.6) | 4243 (86.3) |  |
| missing | 9 (3.3) | 145 (2.9) |  |
| **Normal karyotype, n (%)** |  |  | 0.749 |
| Yes | 146 (54.3) | 2622 (53.3) |  |
| No | 114 (42.4) | 2150 (43.7) |  |
| missing | 9 (3.3) | 145 (2.9) |  |
| **Laboratory, median (IQR)** |  |  |  |
| WBC (10^9^/l) | 7.3 (2.1-34.1) | 18.3 (4.4-57.8) | **<0.001** |
| HB (mmol/l) | 5.6 (5.0-6.6) | 5.8 (5.0-6.6) | 0.498 |
| PLT (10^9^/l) | 60 (33-108) | 55 (30-102) | 0.279 |
| PBB (%) | 26 (8-62) | 38 (10-73) | **0.010** |
| BMB (%) | 66 (43-82) | 70 (44-85) | 0.141 |

**Table S5 Baseline patient characteristics with respect to *BCOR* mutation status.**Abbreviations: acute myeloid leukemia (AML), secondary AML (sAML), therapy-associated AML (tAML), bone marrow blasts (BMB), hemoglobin (HB), interquartile range (IQR), number (n/N), peripheral blood blasts (PBB), platelet count (PLT), white blood cell count (WBC). Boldface indicates statistical significance (*p*<0.05).

| **Variable** | ***EZH2* mutated** | ***EZH2* wildtype** | ***p*** |
| --- | --- | --- | --- |
| **n/N (%)** | 202/5311 (3.8) | 4984/5311 (93.8) |  |
| missing | 125/5311 (2.4) | |  |
| **Age (years), median (IQR)** | 59 (51-66) | 55 (44-64) | **<0.001** |
| **Sex, n (%)** |  |  | 0.001 |
| female | 72 (35.6) | 2389 (47.9) |  |
| male | 130 (64.4) | 2595 (52.1) |  |
| **Disease status, n (%)** |  |  |  |
| de novo | 151 (74.8) | 4204 (84.3) | **<0.001** |
| sAML | 38 (18.8) | 515 (10.3) | **<0.001** |
| tAML | 12 (5.9) | 227 (4.6) | 0.389 |
| missing | 1 (0.5) | 38 (0.7) |  |
| **Complex karyotype, n (%)** |  |  | **0.007** |
| Yes | 10 (5.0) | 538 (10.8) |  |
| No | 185 (91.5) | 4299 (86.3) |  |
| missing | 7 (3.5) | 147 (2.9) |  |
| **Normal karyotype, n (%)** |  |  | 0.240 |
| Yes | 99 (49.0) | 2669 (53.5) |  |
| No | 96 (47.5) | 2168 (43.5) |  |
| missing | 7 (3.5) | 147 (2.9) |  |
| **Laboratory, median (IQR)** |  |  |  |
| WBC (10^9^/l) | 11.3 (2.9-42.9) | 18.0 (4.3-57.3) | **<0.001** |
| HB (mmol/l) | 5.7 (4.9-6.6) | 5.8 (5.0-6.6) | 0.498 |
| PLT (10^9^/l) | 48 (28-98) | 56 (30-103) | 0.279 |
| PBB (%) | 25 (5-56) | 38 (10-73) | **0.010** |
| BMB (%) | 58 (36-80) | 70 (45-85) | 0.141 |

**Table S6 Baseline patient characteristics with respect to *EZH2* mutation status.**Abbreviations: acute myeloid leukemia (AML), secondary AML (sAML), therapy-associated AML (tAML), bone marrow blasts (BMB), hemoglobin (HB), interquartile range (IQR), number (n/N), peripheral blood blasts (PBB), platelet count (PLT), white blood cell count (WBC). Boldface indicates statistical significance (*p*<0.05).

| **Variable** | ***SF3B1* mutated** | ***SF3B1* wildtype** | ***p*** |
| --- | --- | --- | --- |
| **n/N (%)** | 164/5311 (3.1) | 5022/5311 (94.5) |  |
| missing | 125/5311 (2.4) | |  |
| **Age (years), median (IQR)** | 60 (52-67) | 55 (44-64) | **<0.001** |
| **Sex, n (%)** |  |  | 0.874 |
| female | 79 (48.2) | 2382 (47.4) |  |
| male | 85 (51.8) | 2640 (52.6) |  |
| **Disease status, n (%)** |  |  |  |
| de novo | 104 (63.4) | 4251 (84.6) | **<0.001** |
| sAML | 49 (29.9) | 504 (10.0) | **<0.001** |
| tAML | 10 (6.1) | 229 (4.6) | 0.342 |
| missing | 1 (0.6) | 38 (0.8) |  |
| **Complex karyotype, n (%)** |  |  | 0.120 |
| Yes | 11 (6.7) | 537 (10.7) |  |
| No | 149 (90.9) | 4335 (86.3) |  |
| missing | 4 (2.4) | 150 (3.0) |  |
| **Normal karyotype, n (%)** |  |  | 0.519 |
| Yes | 84 (51.2) | 2684 (53.4) |  |
| No | 76 (46.3) | 2188 (43.6) |  |
| missing | 4 (2.4) | 150 (3.0) |  |
| **Laboratory, median (IQR)** |  |  |  |
| WBC (10^9^/l) | 12.9 (4.5-42.7) | 18.0 (4.2-57.3) | 0.332 |
| HB (mmol/l) | 5.6 (4.8-6.5) | 5.8 (5.0-6.6) | 0.098 |
| PLT (10^9^/l) | 72 (36-134) | 55 (30-101) | **0.002** |
| PBB (%) | 23 (6-47) | 38 (10-73) | **<0.001** |
| BMB (%) | 50 (35-74) | 70 (45-85) | **<0.001** |

**Table S7 Baseline patient characteristics with respect to *SF3B1* mutation status.**Abbreviations: acute myeloid leukemia (AML), secondary AML (sAML), therapy-associated AML (tAML), bone marrow blasts (BMB), hemoglobin (HB), interquartile range (IQR), number (n/N), peripheral blood blasts (PBB), platelet count (PLT), white blood cell count (WBC). Boldface indicates statistical significance (*p*<0.05).

| **Variable** | ***SRSF2* mutated** | ***SRSF2* wildtype** | ***p*** |
| --- | --- | --- | --- |
| **n/N (%)** | 459/5311 (8.6) | 4727/5311 (89.0) |  |
| missing | 125/5311 (2.4) | |  |
| **Age (years), median (IQR)** | 63 (57-69) | 54 (43-63) | **<0.001** |
| **Sex, n (%)** |  |  | **<0.001** |
| female | 117 (25.5) | 2344 (49.6) |  |
| male | 342 (74.5) | 2383 (50.4) |  |
| **Disease status, n (%)** |  |  |  |
| de novo | 345 (75.2) | 4010 (84.8) | **<0.001** |
| sAML | 96 (20.9) | 457 (9.7) | **<0.001** |
| tAML | 14 (3.0) | 225 (4.8) | 0.102 |
| missing | 4 (0.9) | 35 (0.7) |  |
| **Complex karyotype, n (%)** |  |  | **<0.001** |
| Yes | 26 (5.7) | 522 (11.0) |  |
| No | 423 (92.1) | 4061 (85.9) |  |
| missing | 10 (2.2) | 144 (3.0) |  |
| **Normal karyotype, n (%)** |  |  | 0.001 |
| Yes | 281 (61.2) | 2487 (52.6) |  |
| No | 168 (36.6) | 2096 (44.3) |  |
| missing | 10 (2.2) | 144 (3.0) |  |
| **Laboratory, median (IQR)** |  |  |  |
| WBC (10^9^/l) | 10.7 (3.0-35.3) | 18.6 (4.4-58.7) | **<0.001** |
| HB (mmol/l) | 5.8 (5.1-6.7) | 5.7 (5.0-6.6) | 0.246 |
| PLT (10^9^/l) | 52 (29-92) | 56 (30-103) | 0.096 |
| PBB (%) | 27 (6-63) | 38 (10-73) | **<0.001** |
| BMB (%) | 61 (35-83) | 70 (45-85) | **<0.001** |

**Table S8 Baseline patient characteristics with respect to *SRSF2* mutation status.**Abbreviations: acute myeloid leukemia (AML), secondary AML (sAML), therapy-associated AML (tAML), bone marrow blasts (BMB), hemoglobin (HB), interquartile range (IQR), number (n/N), peripheral blood blasts (PBB), platelet count (PLT), white blood cell count (WBC). Boldface indicates statistical significance (*p*<0.05).

| **Variable** | ***STAG2* mutated** | ***STAG2* wildtype** | ***p*** |
| --- | --- | --- | --- |
| **n/N (%)** | 293/5311 (5.5) | 4619/5311 (87.0) |  |
| missing | 399/5311 (7.5) | |  |
| **Age (years), median (IQR)** | 60 (52-67) | 55 (44-64) | **<0.001** |
| **Sex, n (%)** |  |  | **<0.001** |
| female | 106 (36.2) | 2225 (48.2) |  |
| male | 187 (63.8) | 2394 (51.8) |  |
| **Disease status, n (%)** |  |  |  |
| de novo | 212 (72.4) | 3930 (85.1) | **<0.001** |
| sAML | 64 (21.8) | 454 (9.8) | **<0.001** |
| tAML | 12 (4.1) | 201 (4.4) | 1.000 |
| missing | 5 (1.7) | 34 (0.7) |  |
| **Complex karyotype, n (%)** |  |  | **<0.001** |
| Yes | 13 (4.4) | 496 (10.7) |  |
| No | 269 (91.8) | 3984 (86.3) |  |
| missing | 11 (3.8) | 139 (3.0) |  |
| **Normal karyotype, n (%)** |  |  | **<0.001** |
| Yes | 204 (69.6) | 2440 (52.8) |  |
| No | 78 (26.6) | 2040 (44.2) |  |
| missing | 11 (3.8) | 139 (3.0) |  |
| **Laboratory, median (IQR)** |  |  |  |
| WBC (10^9^/l) | 6.2 (2.2-26.0) | 19.7 (4.8-59.8) | **<0.001** |
| HB (mmol/l) | 5.6 (4.9-6.5) | 5.8 (5.0-6.6) | 0.369 |
| PLT (10^9^/l) | 48 (27-85) | 55 (30-102) | **0.016** |
| PBB (%) | 23 (4-67) | 38 (10-73) | **<0.001** |
| BMB (%) | 54 (31-80) | 70 (46-85) | **<0.001** |

**Table S9 Baseline patient characteristics with respect to *STAG2* mutation status.**Abbreviations: acute myeloid leukemia (AML), secondary AML (sAML), therapy-associated AML (tAML), bone marrow blasts (BMB), hemoglobin (HB), interquartile range (IQR), number (n/N), peripheral blood blasts (PBB), platelet count (PLT), white blood cell count (WBC). Boldface indicates statistical significance (*p*<0.05).

| **Variable** | ***U2AF1* mutated** | ***U2AF1* wildtype** | ***p*** |
| --- | --- | --- | --- |
| **n/N (%)** | 140/5311 (2.6) | 4772/5311 (89.9) |  |
| missing | 399/5311 (7.5) | |  |
| **Age (years), median (IQR)** | 63 (56-69) | 55 (44-64) | **<0.001** |
| **Sex, n (%)** |  |  | **<0.001** |
| female | 40 (28.6) | 2291 (48.0) |  |
| male | 100 (71.4) | 2481 (52.0) |  |
| **Disease status, n (%)** |  |  |  |
| de novo | 96 (68.6) | 4046 (84.8) | **<0.001** |
| sAML | 41 (29.3) | 477 (10.0) | **<0.001** |
| tAML | 2 (1.4) | 211 (4.4) | 0.092 |
| missing | 1 (0.7) | 38 (0.8) |  |
| **Complex karyotype, n (%)** |  |  | 0.398 |
| Yes | 11 (7.8) | 498 (10.4) |  |
| No | 125 (89.3) | 4128 (86.5) |  |
| missing | 4 (2.9) | 146 (3.1) |  |
| **Normal karyotype, n (%)** |  |  | 0.054 |
| Yes | 64 (45.7) | 2580 (54.0) |  |
| No | 72 (51.4) | 2046 (42.9) |  |
| missing | 4 (2.9) | 146 (3.1) |  |
| **Laboratory, median (IQR)** |  |  |  |
| WBC (10^9^/l) | 6.9 (2.5-36.6) | 18.7 (4.6-57.9) | **<0.001** |
| HB (mmol/l) | 5.6 (4.9-6.4) | 5.7 (5.0-6.6) | 0.488 |
| PLT (10^9^/l) | 53 (31-107) | 55 (30-101) | 0.998 |
| PBB (%) | 14 (2-52) | 38 (10-73) | **<0.001** |
| BMB (%) | 57 (30-80) | 70 (45-85) | **<0.001** |

**Table S10 Baseline patient characteristics with respect to *U2AF1* mutation status.**Abbreviations: acute myeloid leukemia (AML), secondary AML (sAML), therapy-associated AML (tAML), bone marrow blasts (BMB), hemoglobin (HB), interquartile range (IQR), number (n/N), peripheral blood blasts (PBB), platelet count (PLT), white blood cell count (WBC). Boldface indicates statistical significance (*p*<0.05).

| **Variable** | ***ZRSR2* mutated** | ***ZRSR2* wildtype** | ***p*** |
| --- | --- | --- | --- |
| **n/N (%)** | 71/5311 (1.3) | 5049/5311 (95.1) |  |
| missing | 191/5311 (3.6) | |  |
| **Age (years), median (IQR)** | 60 (48-66) | 55 (44-64) | **0.016** |
| **Sex, n (%)** |  |  | **<0.001** |
| female | 19 (26.8) | 2419 (47.9) |  |
| male | 52 (73.2) | 2630 (52.1) |  |
| **Disease status, n (%)** |  |  |  |
| de novo | 59 (83.1) | 4245 (84.1) | 1.000 |
| sAML | 9 (12.7) | 538 (10.7) | 0.555 |
| tAML | 1 (1.4) | 229 (4.5) | 0.374 |
| missing | 2 (2.8) | 37 (0.7) |  |
| **Complex karyotype, n (%)** |  |  | 0.340 |
| Yes | 10 (14.1) | 529 (10.5) |  |
| No | 61 (85.9) | 4367 (86.5) |  |
| missing | 0 | 153 (3.0) |  |
| **Normal karyotype, n (%)** |  |  | 0.093 |
| Yes | 32 (45.1) | 2707 (53.6) |  |
| No | 39 (54.9) | 2189 (43.4) |  |
| missing | 0 | 153 (3.0) |  |
| **Laboratory, median (IQR)** |  |  |  |
| WBC (10^9^/l) | 20.0 (3.9-58.6) | 17.9 (4.3-56.7) | 0.840 |
| HB (mmol/l) | 5.9 (5.0-7.1) | 5.8 (5.0-6.6) | 0.392 |
| PLT (10^9^/l) | 53 (28-105) | 55 (30-101) | 0.808 |
| PBB (%) | 29 (6-61) | 37 (10-73) | 0.220 |
| BMB (%) | 57 (38-85) | 70 (45-85) | 0.117 |

**Table S11 Baseline patient characteristics with respect to *ZRSZ2* mutation status.**Abbreviations: acute myeloid leukemia (AML), secondary AML (sAML), therapy-associated AML (tAML), bone marrow blasts (BMB), hemoglobin (HB), interquartile range (IQR), number (n/N), peripheral blood blasts (PBB), platelet count (PLT), white blood cell count (WBC). Boldface indicates statistical significance (*p*<0.05).

| **Variable** | ***RUNX1* mutated** | ***RUNX1* wildtype** | ***p*** |
| --- | --- | --- | --- |
| **n/N (%)** | 638/5311 (12.0) | 4548/5311 (86.3) |  |
| missing | 125/5311 (2.4) | |  |
| **Age (years), median (IQR)** | 61 (51-68) | 54 (44-63) | **<0.001** |
| **Sex, n (%)** |  |  | **<0.001** |
| female | 252 (39.5) | 2209 (48.6) |  |
| male | 386 (60.5) | 2339 (51.4) |  |
| **Disease status, n (%)** |  |  |  |
| de novo | 463 (72.6) | 3892 (85.6) | **<0.001** |
| sAML | 141 (22.1) | 412 (9.0) | **<0.001** |
| tAML | 30 (4.7) | 209 (4.6) | 0.920 |
| missing | 4 (0.6) | 35 (0.8) |  |
| **Complex karyotype, n (%)** |  |  | 0.073 |
| Yes | 54 (8.5) | 494 (10.9) |  |
| No | 564 (88.4) | 3920 (86.2) |  |
| missing | 20 (3.1) | 134 (2.9) |  |
| **Normal karyotype, n (%)** |  |  | 0.575 |
| Yes | 333 (52.2) | 2435 (53.5) |  |
| No | 285 (44.7) | 1979 (43.5) |  |
| missing | 20 (3.1) | 134 (2.9) |  |
| **Laboratory, median (IQR)** |  |  |  |
| WBC (10^9^/l) | 12.9 (3.3-43.0) | 18.6 (4.4-58.3) | **<0.001** |
| HB (mmol/l) | 5.5 (4.8-6.4) | 5.8 (5.0-6.7) | **<0.001** |
| PLT (10^9^/l) | 56 (31-108) | 55 (30-101) | 0.429 |
| PBB (%) | 28 (7-65) | 38 (10-74) | **<0.001** |
| BMB (%) | 66 (40-84) | 70 (45-85) | **0.003** |

**Table S12 Baseline patient characteristics with respect to *RUNX1* mutation status.**Abbreviations: acute myeloid leukemia (AML), secondary AML (sAML), therapy-associated AML (tAML), bone marrow blasts (BMB), hemoglobin (HB), interquartile range (IQR), number (n/N), peripheral blood blasts (PBB), platelet count (PLT), white blood cell count (WBC). Boldface indicates statistical significance (*p*<0.05).

| **Outcome** | **MR gene mutation** | **no MR gene mutation** | **OR/HR** | ***p*** |
| --- | --- | --- | --- | --- |
| **patients who underwent allo HSCT in first complete remission** | | | | |
| **EFS** | 26.5 [19.0-42.9] | 50.5 [28.1-101.1] | 1.19 [1.00-1.42] | 0.053 |
| **RFS** | 41.1 [26.8-68.3] | 101.9 [51.2-n.a.] | 1.19 [0.98-1.43] | 0.072 |
| **OS** | 63.4 [50.9-89.7] | n.r. [101.1-n.a.] | 1.23 [1.02-1.50] | **0.033** |
| **patients who did not undergo allo HSCT in first complete remission** | | | | |
| **EFS** | 3.8 [3.3-4.8] | 8.6 [8.0-9.4] | 1.55 [1.44-1.66] | **<0.001** |
| **RFS** | 11.1 [9.8-12.5] | 15.5 [14.4-17.1] | 1.43 [1.30-1.58] | **<0.001** |
| **OS** | 13.6 [12.6-14.7] | 21.3 [19.1-23.8] | 1.51 [1.39-1.63] | **<0.001** |

**Table S13 Summary of patient outcomes in context of allogeneic stem cell transplantation.**Survival times are displayed in months. Square brackets show 95%-confidence intervals. Boldface indicates statistical significance (p<0.05) Abbreviations: event-free survival (EFS), hazard ratio (HR), number (n/N), not available (n.a.), not reached (n.r.), overall survival (OS), relapse-free-survival (RFS).

| **Outcome** | **mut. *ASXL1*** | **wt. *ASXL1*** | **OR/HR** | ***p*** |
| --- | --- | --- | --- | --- |
| n/N (%) | 494/5311 (9.3) | 4692/5311 (88.3) |  |  |
| missing | 125/5311 (2.4) | |  |  |
| CR rate, n (%) | 294/494 (59.5) | 3539/4692 (75.4) | 0.48 [0.40-0.58] | **<0.001** |
| EFS | 6.0 [4.7-7.6] | 9.4 [8.9-9.9] | 1.40 [1.27-1.55] | **<0.001** |
| RFS | 14.3 [11.6-17.3] | 18.0 [16.6-20.0] | 1.33 [1.16-1.53] | **<0.001** |
| OS | 15.3 [13.2-17.2] | 23.1 [21.5-25.3] | 1.51 [1.36-1.68] | **<0.001** |

**Table S14 Summary of patient outcome with respect to *ASXL1* mutation status.** Survival is reported for both the WHO and ICC definitions of SMT. Survival times are displayed in months. Square brackets show 95%-confidence intervals. Boldface indicates statistical significance (p<0.05). Abbreviations: complete remission (CR), event-free survival (EFS), hazard ratio (HR), mutated (mut.), number (n/N), odds ratio (OR), overall survival (OS), relapse-free-survival (RFS), wild-type (wt).

| **Outcome** | **mut. *BCOR*** | **wt. *BCOR*** | **OR/HR** | ***p*** |
| --- | --- | --- | --- | --- |
| **n/N (%)** | 269/5311 (5.0) | 4917/5311 (92.6) |  |  |
| **missing** | 125/5311 (2.4) | |  |  |
| **CR rate, n (%)** | 178/269 (66.2) | 3655/4917 (74.3) | 0.68 [0.52-0.88] | **0.003** |
| **EFS** | 6.2 [3.6-8.5] | 9.2 [8.7-9.7] | 1.38 [1.21-1.58] | **<0.001** |
| **RFS** | 15.0 [9.9-19.0] | 17.8 [16.4-19.6] | 1.28 [1.07-1.52] | **0.007** |
| **OS** | 16.5 [12.5-21.1] | 22.2 [20.8-24.0] | 1.26 [1.09-1.46] | **0.002** |

**Table S15 Summary of patient outcome with respect to *BCOR* mutation status.** Survival is reported for both the WHO and ICC definitions of SMT. Survival times are displayed in months. Square brackets show 95%-confidence intervals. Boldface indicates statistical significance (p<0.05). Abbreviations: complete remission (CR), event-free survival (EFS), hazard ratio (HR), mutated (mut.), number (n/N), odds ratio (OR), overall survival (OS), relapse-free-survival (RFS), wild-type (wt).

| **Outcome** | **mut. *EZH2*** | **wt. *EZH2*** | **OR/HR** | ***p*** |
| --- | --- | --- | --- | --- |
| **n/N (%)** | 202/5311 (3.8) | 4984/5311 (93.8) |  |  |
| **missing** | 125/5311 (2.4) | |  |  |
| **CR rate, n (%)** | 144/202 (71.3) | 3689/4984 (74.0) | 0.87 [0.64-1.29] | 0.387 |
| **EFS** | 8.5 [6.4-10.6] | 9.1 [8.6-9.6] | 1.11 [0.95-1.30] | 0.167 |
| **RFS** | 16.6 [11.2-23.4] | 17.7 [16.3-19.2] | 1.16 [0.95-1.41] | 0.149 |
| **OS** | 19.8 [14.7-24.2] | 22.0 [20.6-23.6] | 1.16 [0.98-1.37] | 0.088 |

**Table S16 Summary of patient outcome with respect to *EZH2* mutation status.** Survival is reported for both the WHO and ICC definitions of SMT. Survival times are displayed in months. Square brackets show 95%-confidence intervals. Boldface indicates statistical significance (p<0.05). Abbreviations: complete remission (CR), event-free survival (EFS), hazard ratio (HR), mutated (mut.), number (n/N), odds ratio (OR), overall survival (OS), relapse-free-survival (RFS), wild-type (wt).

| **Outcome** | **mut. *SF3B1*** | **wt. *SF3B1*** | **OR/HR** | ***p*** |
| --- | --- | --- | --- | --- |
| **n/N (%)** | 164/5311 (3.1) | 5022/5311 (94.5) |  |  |
| **missing** | 125/5311 (2.4) | |  |  |
| **CR rate, n (%)** | 96/164 (58.5) | 3737/5022 (74.4) | 0.49 [0.35-0.67] | **<0.001** |
| **EFS** | 3.0 [2.1-4.7] | 9.3 [8.8-9.8] | 1.70 [1.44-2.01] | **<0.001** |
| **RFS** | 11.7 [7.1-14.7] | 17.9 [16.5-19.6] | 1.40 [1.11-1.78] | **0.005** |
| **OS** | 13.7 [10.5-14.5] | 22.4 [21.0-24.3] | 1.56 [1.31-1.86] | **<0.001** |

**Table S17 Summary of patient outcome with respect to *SF3B1* mutation status.** Survival is reported for both the WHO and ICC definitions of SMT. Survival times are displayed in months. Square brackets show 95%-confidence intervals. Boldface indicates statistical significance (p<0.05). Abbreviations: complete remission (CR), event-free survival (EFS), hazard ratio (HR), mutated (mut.), number (n/N), odds ratio (OR), overall survival (OS), relapse-free-survival (RFS), wild-type (wt).

| **Outcome** | **mut. *SRSF2*** | **wt. *SRSF2*** | **OR/HR** | ***p*** |
| --- | --- | --- | --- | --- |
| **n/N (%)** | 459/5311 (8.6) | 4727/5311 (89.0) |  |  |
| **missing** | 125/5311 (2.4) | |  |  |
| **CR rate, n (%)** | 265/459 (57.7) | 3568/4727 (75.5) | 0.44 [0.36-0.54] | **<0.001** |
| **EFS** | 5.0 [3.4-6.5] | 9.5 [9.0-10.0] | 1.46 [1.32-1.62] | **<0.001** |
| **RFS** | 15.4 [11.9-18.2] | 17.9 [16.4-19.9] | 1.32 [1.14-1.53] | **<0.001** |
| **OS** | 16.0 [13.1-18.7] | 22.7 [21.2-25.0] | 1.49 [1.33-1.66] | **<0.001** |

**Table S18 Summary of patient outcome with respect to *SRSF2* mutation status.** Survival is reported for both the WHO and ICC definitions of SMT. Survival times are displayed in months. Square brackets show 95%-confidence intervals. Boldface indicates statistical significance (p<0.05). Abbreviations: complete remission (CR), event-free survival (EFS), hazard ratio (HR), mutated (mut.), number (n/N), odds ratio (OR), overall survival (OS), relapse-free-survival (RFS), wild-type (wt).

| **Outcome** | **mut. *STAG2*** | **wt. *STAG2*** | **OR/HR** | ***p*** |
| --- | --- | --- | --- | --- |
| **n/N (%)** | 293/5311 (5.5) | 4619/5311 (87.0) |  |  |
| **missing** | 399/5311 (7.5) | |  |  |
| **CR rate, n (%)** | 181/293 (61.8) | 3424/4619 (74.1) | 0.56 [0.44-0.72] | **<0.001** |
| **EFS** | 6.2 [4.2-9.4] | 9.0 [8.5-9.5] | 1.17 [1.03-1.34] | **0.017** |
| **RFS** | 23.5 [15.7-34.5] | 17.1 [15.8-18.8] | 0.93 [0.77-1.12] | 0.433 |
| **OS** | 19.4 [15.3-26.7] | 21.5 [20.1-22.9] | 1.12 [0.97-1.30] | 0.108 |

**Table S19 Summary of patient outcome with respect to *STAG2* mutation status.** Survival is reported for both the WHO and ICC definitions of SMT. Survival times are displayed in months. Square brackets show 95%-confidence intervals. Boldface indicates statistical significance (p<0.05). Abbreviations: complete remission (CR), event-free survival (EFS), hazard ratio (HR), mutated (mut.), number (n/N), odds ratio (OR), overall survival (OS), relapse-free-survival (RFS), wild-type (wt).

| **Outcome** | **mut. *U2AF1*** | **wt. *U2AF1*** | **OR/HR** | ***p*** |
| --- | --- | --- | --- | --- |
| **n/N (%)** | 140/5311 (2.6) | 4772/5311 (89.9) |  |  |
| **missing** | 399/5311 (7.5) | |  |  |
| **CR rate, n (%)** | 73/140 (52.1) | 3532/4772 (74.0) | 0.38 [0.27-0.54] | **<0.001** |
| **EFS** | 3.3 [2.5-6.4] | 9.1 [8.5-9.6] | 1.79 [1.50-2.13] | **<0.001** |
| **RFS** | 9.9 [7.3-11.8] | 17.9 [16.4-19.6] | 1.88 [1.46-2.41] | **<0.001** |
| **OS** | 12.4 [9.6-14.6] | 22.0 [20.7-23.7] | 1.87 [1.56-2.24] | **<0.001** |

**Table S20 Summary of patient outcome with respect to *U2AF1* mutation status.** Survival is reported for both the WHO and ICC definitions of SMT. Survival times are displayed in months. Square brackets show 95%-confidence intervals. Boldface indicates statistical significance (p<0.05). Abbreviations: complete remission (CR), event-free survival (EFS), hazard ratio (HR), mutated (mut.), number (n/N), odds ratio (OR), overall survival (OS), relapse-free-survival (RFS), wild-type (wt).

| **Outcome** | **mut. *ZRSR2*** | **wt. *ZRSR2*** | **OR/HR** | ***p*** |
| --- | --- | --- | --- | --- |
| **n/N (%)** | 71/5311 (1.3) | 5049/5311 (95.1) |  |  |
| **missing** | 191/5311 (3.6) | |  |  |
| **CR rate, n (%)** | 52/71 (73.2) | 3737/5049 (74.0) | 0.96 [0.57-1.63] | 0.882 |
| **EFS** | 5.3 [3.0-10.9] | 9.1 [8.6-9.6] | 1.17 [0.90-1.53] | 0.232 |
| **RFS** | 13.7 [6.5-39.8] | 17.7 [16.3-19.1] | 1.19 [0.86-1.67] | 0.297 |
| **OS** | 17.6 [10.2-27.0] | 22.0 [20.7-23.6] | 1.28 [0.97-1.68] | 0.084 |

**Table S21 Summary of patient outcome with respect to *ZRSR2* mutation status.** Survival is reported for both the WHO and ICC definitions of SMT. Survival times are displayed in months. Square brackets show 95%-confidence intervals. Boldface indicates statistical significance (p<0.05). Abbreviations: complete remission (CR), event-free survival (EFS), hazard ratio (HR), mutated (mut.), number (n/N), odds ratio (OR), overall survival (OS), relapse-free-survival (RFS), wild-type (wt).

| **Outcome** | **mut. *RUNX1*** | **wt. *RUNX1*** | **OR/HR** | ***p*** |
| --- | --- | --- | --- | --- |
| **n/N (%)** | 638/5311 (12.0) | 4548/5311 (86.3) |  |  |
| **missing** | 125/5311 (2.4) | |  |  |
| **CR rate, n (%)** | 390/638 (61.1) | 3443/4548 (75.7) | 0.50 [0.42-0.60] | **<0.001** |
| **EFS** | 5.2 [3.9-6.6] | 9.6 [9.1-10.2] | 1.53 [1.40-1.67] | **<0.001** |
| **RFS** | 12.2 [10.1-14.2] | 18.8 [17.2-21.0] | 1.45 [1.28-1.63] | **<0.001** |
| **OS** | 14.5 [12.9-17.1] | 24.0 [22.0-26.0] | 1.52 [1.38-1.67] | **<0.001** |

**Table S22 Summary of patient outcome with respect to *RUNX1* mutation status.** Survival is reported for both the WHO and ICC definitions of SMT. Survival times are displayed in months. Square brackets show 95%-confidence intervals. Boldface indicates statistical significance (p<0.05). Abbreviations: complete remission (CR), event-free survival (EFS), hazard ratio (HR), mutated (mut.), number (n/N), odds ratio (OR), overall survival (OS), relapse-free-survival (RFS), wild-type (wt).


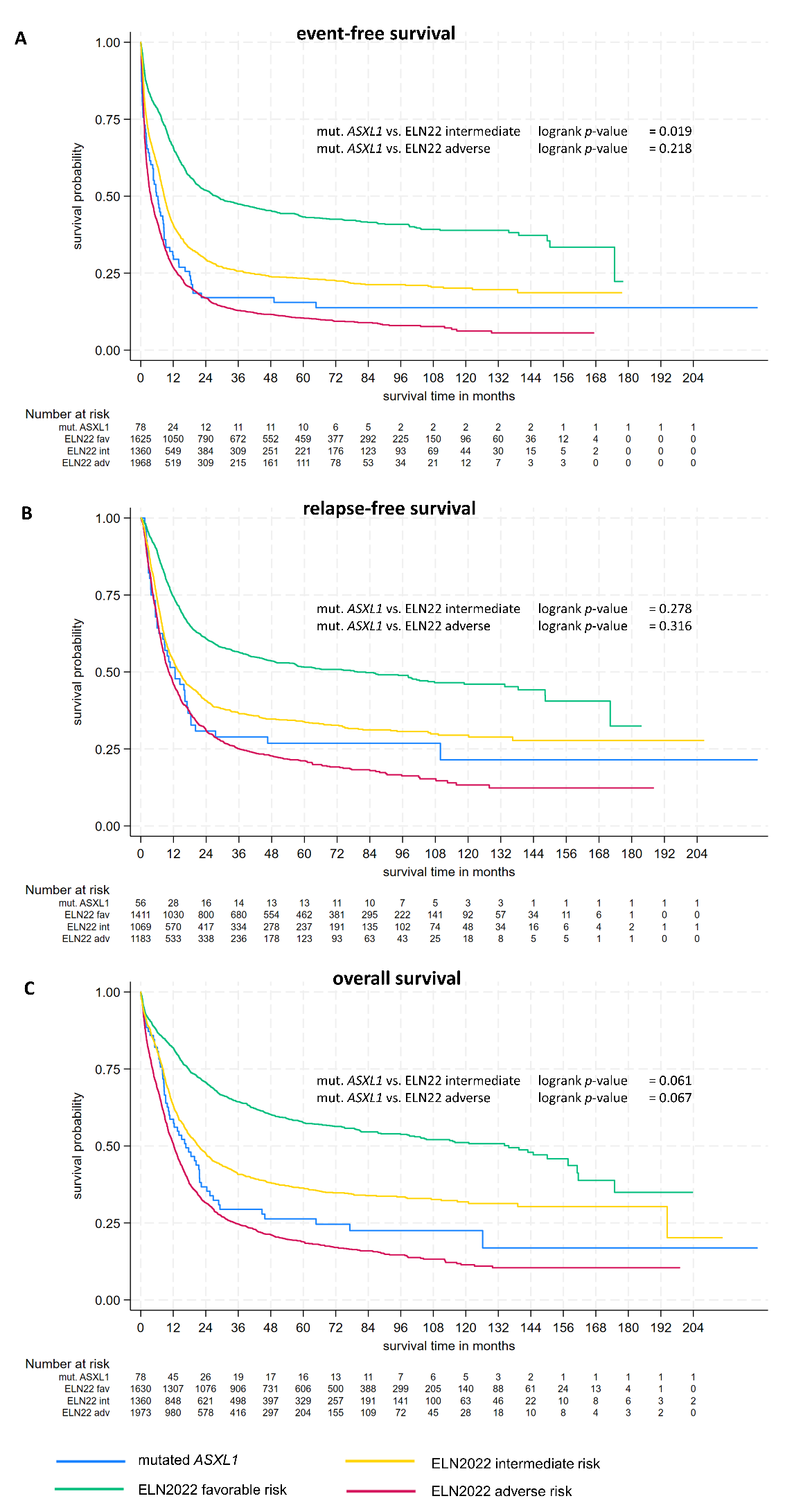


**Figure S1 Kaplan-Meier plots comparing ELN2022 risk groups to AML with altered *ASXL1*.** Event-free survival (EFS, A), relapse-free survival (RFS, B), and overall survival (OS, C) are shown. Patients from the entire cohort were retrospectively assigned to ELN2022 risk groups. Patients within the ELN2022 adverse risk group that had altered *ASXL1* were treated as a separate group for this Kaplan-Meier analysis. Patients with *ASXL1* and co-occurring class-defining favorable or intermediate risk features were treated as favorable or intermediate risk, respectively.


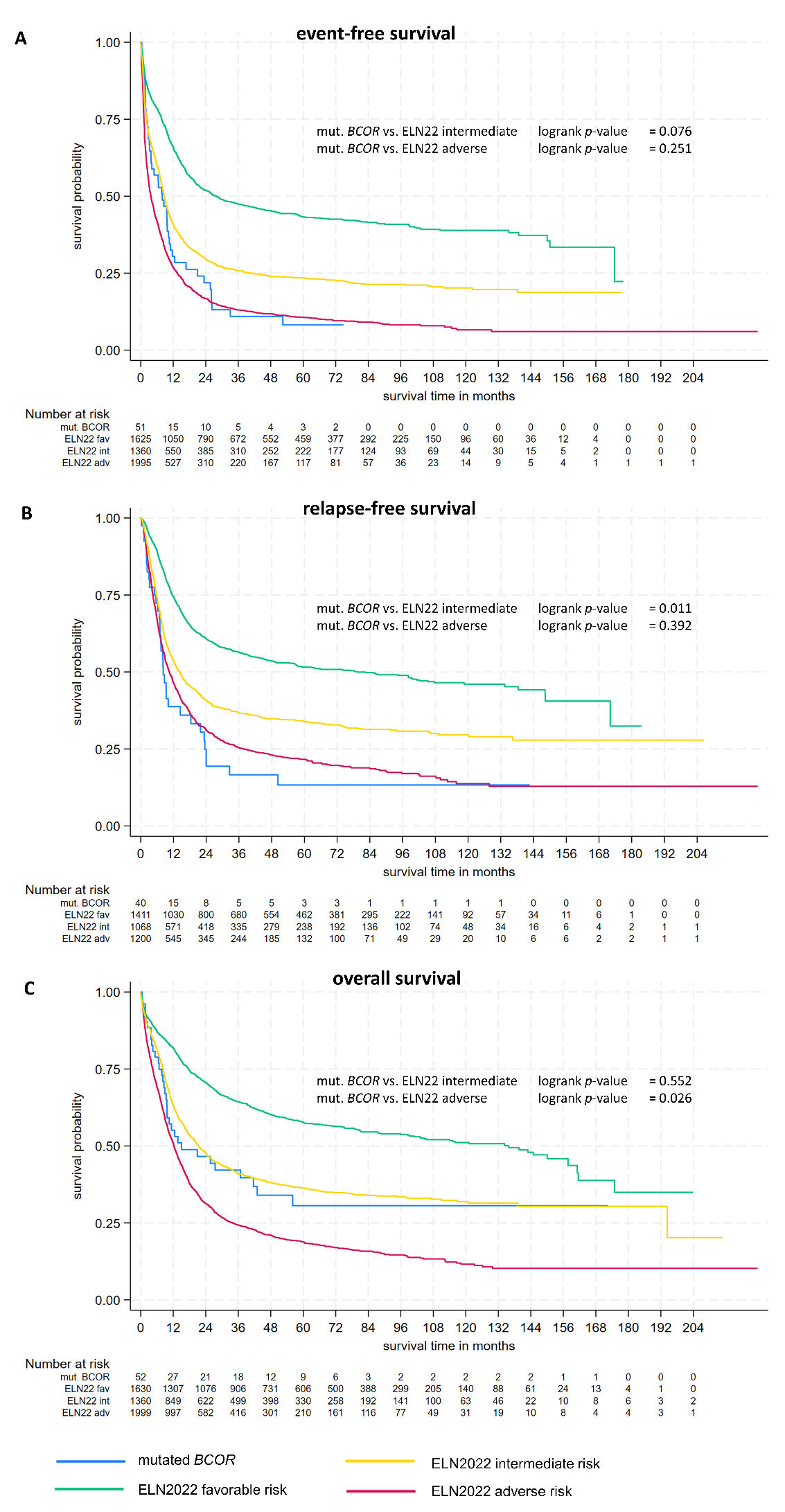


**Figure S2 Kaplan-Meier plots comparing ELN2022 risk groups to AML with altered *BCOR*.** Event-free survival (EFS, A), relapse-free survival (RFS, B), and overall survival (OS, C) are shown. Patients from the entire cohort were retrospectively assigned to ELN2022 risk groups. Patients within the ELN2022 adverse risk group that had altered *BCOR* were treated as a separate group for this Kaplan-Meier analysis. Patients with *BCOR* and co-occurring class-defining favorable or intermediate risk features were treated as favorable or intermediate risk, respectively.


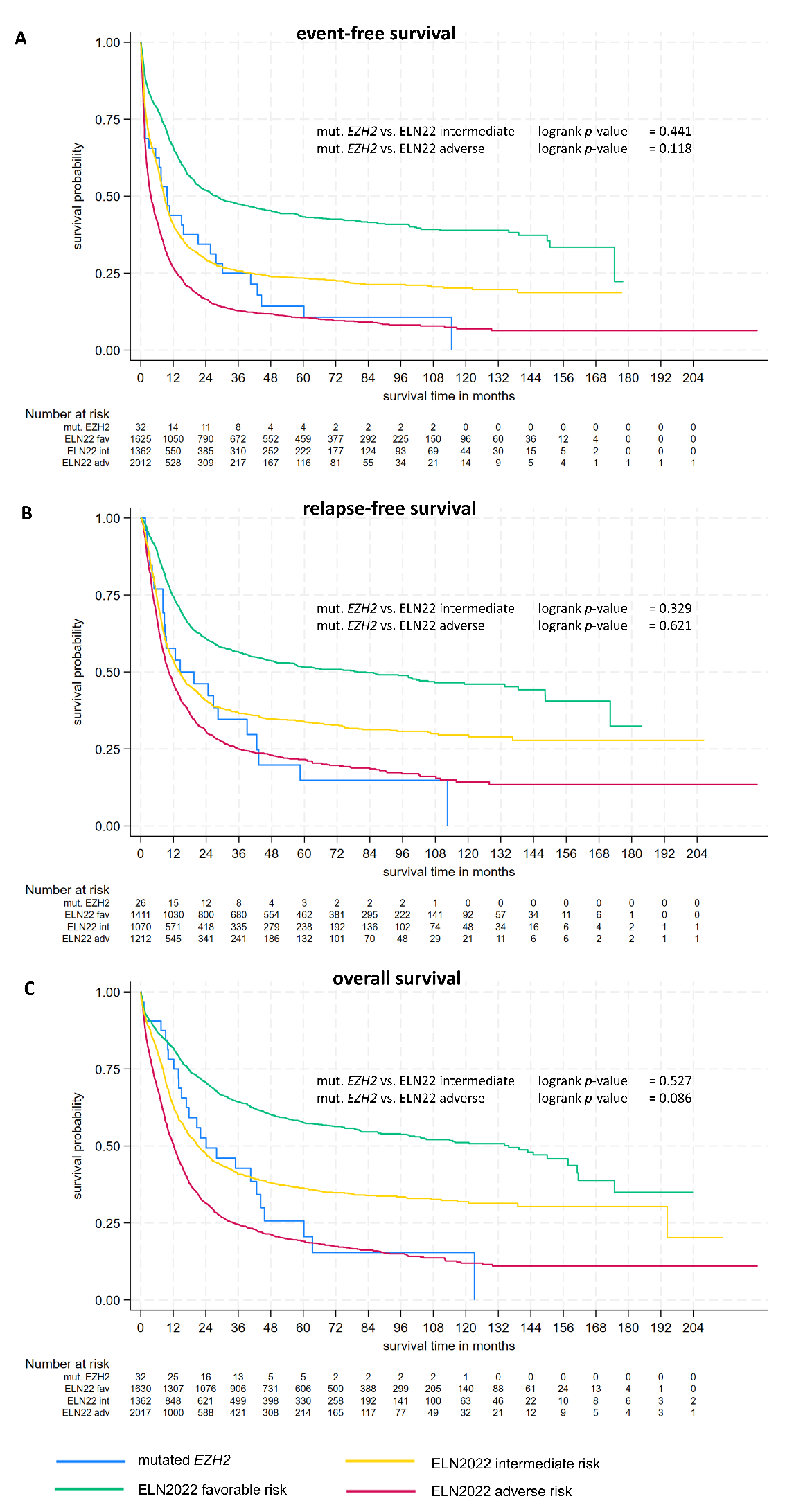


**Figure S3 Kaplan-Meier plots comparing ELN2022 risk groups to AML with altered *EZH2*.** Event-free survival (EFS, A), relapse-free survival (RFS, B), and overall survival (OS, C) are shown. Patients from the entire cohort were retrospectively assigned to ELN2022 risk groups. Patients within the ELN2022 adverse risk group that had altered *EZH2* were treated as a separate group for this Kaplan-Meier analysis. Patients with *EZH2* and co-occurring class-defining favorable or intermediate risk features were treated as favorable or intermediate risk, respectively.


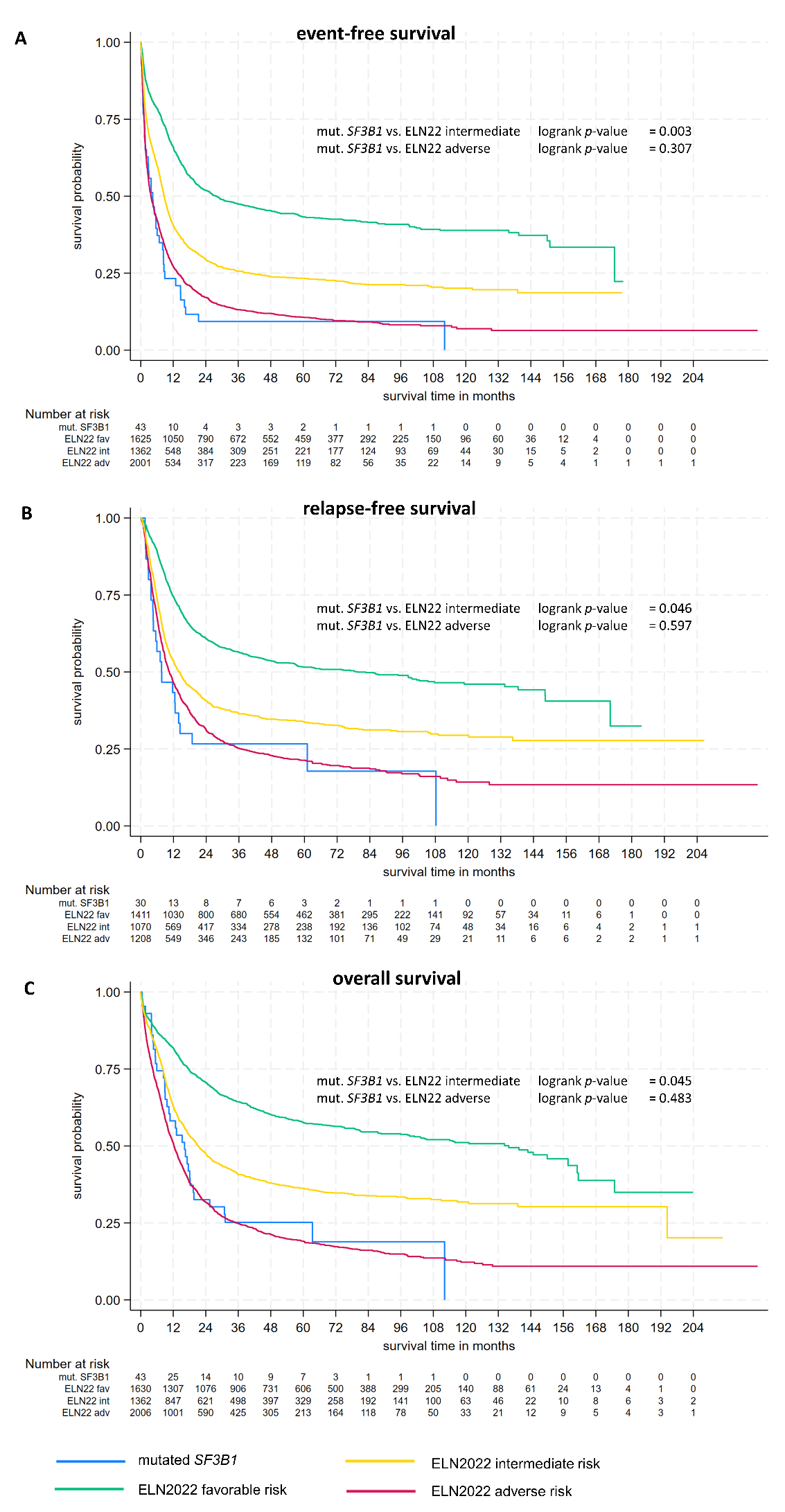


**Figure S4 Kaplan-Meier plots comparing ELN2022 risk groups to AML with altered *SF3B1*.** Event-free survival (EFS, A), relapse-free survival (RFS, B), and overall survival (OS, C) are shown. Patients from the entire cohort were retrospectively assigned to ELN2022 risk groups. Patients within the ELN2022 adverse risk group that had altered *SF3B1* were treated as a separate group for this Kaplan-Meier analysis. Patients with *SF3B1* and co-occurring class-defining favorable or intermediate risk features were treated as favorable or intermediate risk, respectively.


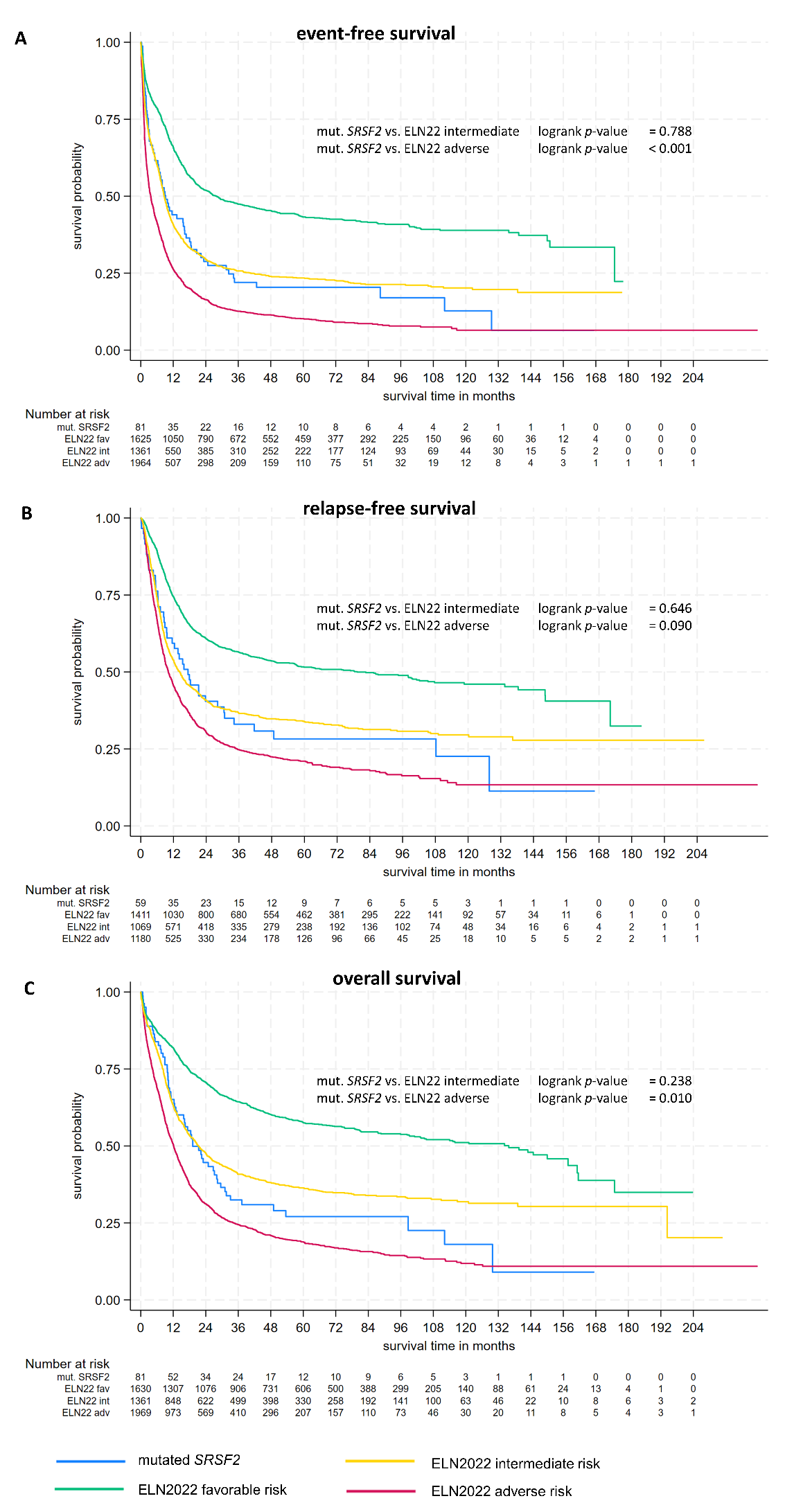


**Figure S5 Kaplan-Meier plots comparing ELN2022 risk groups to AML with altered *SRSF2*.** Event-free survival (EFS, A), relapse-free survival (RFS, B), and overall survival (OS, C) are shown. Patients from the entire cohort were retrospectively assigned to ELN2022 risk groups. Patients within the ELN2022 adverse risk group that had altered *SRSF2* were treated as a separate group for this Kaplan-Meier analysis. Patients with *SRSF2* and co-occurring class-defining favorable or intermediate risk features were treated as favorable or intermediate risk, respectively.


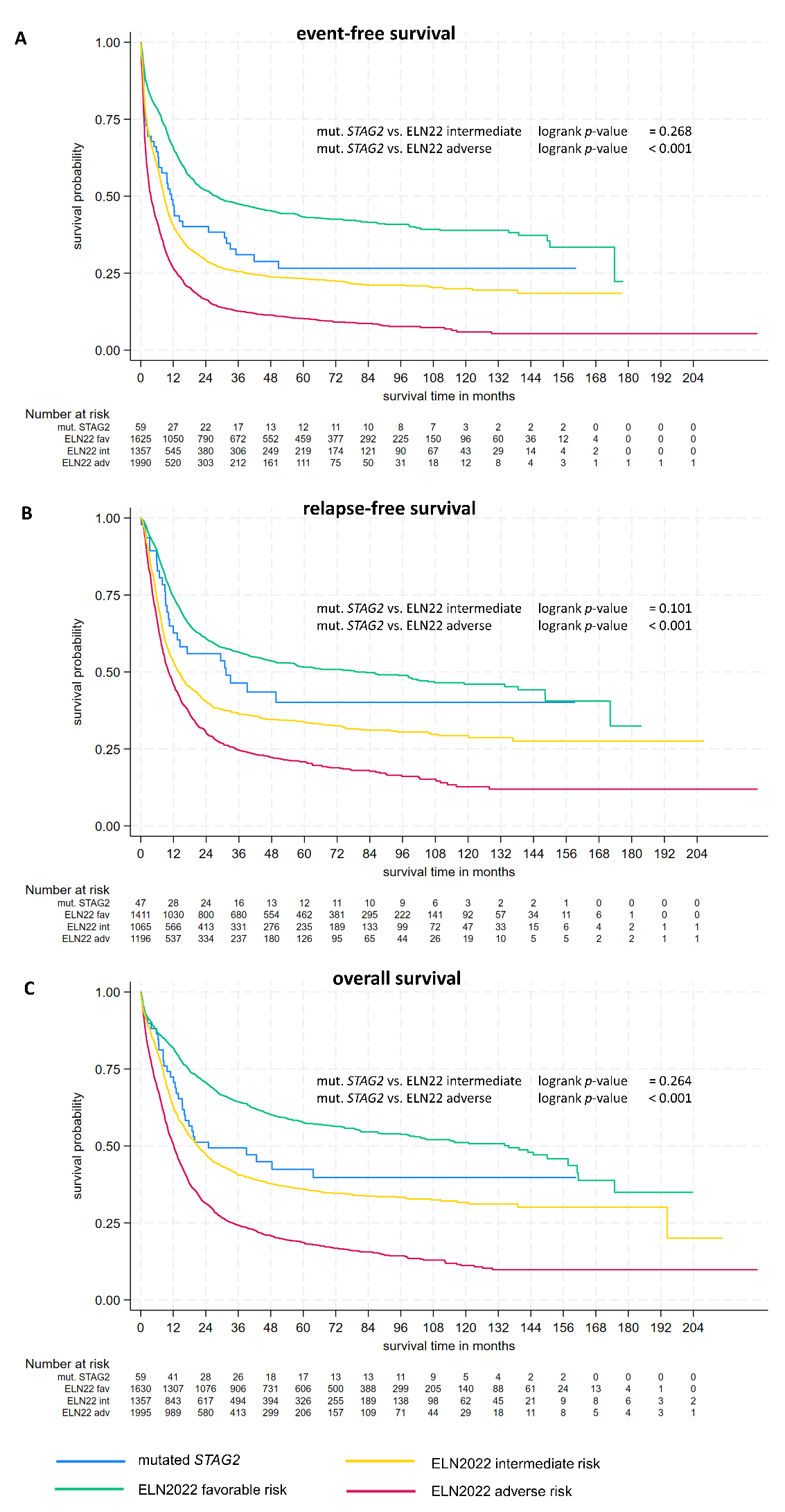


**Figure S6 Kaplan-Meier plots comparing ELN2022 risk groups to AML with altered *STAG2*.** Event-free survival (EFS, A), relapse-free survival (RFS, B), and overall survival (OS, C) are shown. Patients from the entire cohort were retrospectively assigned to ELN2022 risk groups. Patients within the ELN2022 adverse risk group that had altered *STAG2* were treated as a separate group for this Kaplan-Meier analysis. Patients with *STAG2* and co-occurring class-defining favorable or intermediate risk features were treated as favorable or intermediate risk, respectively.


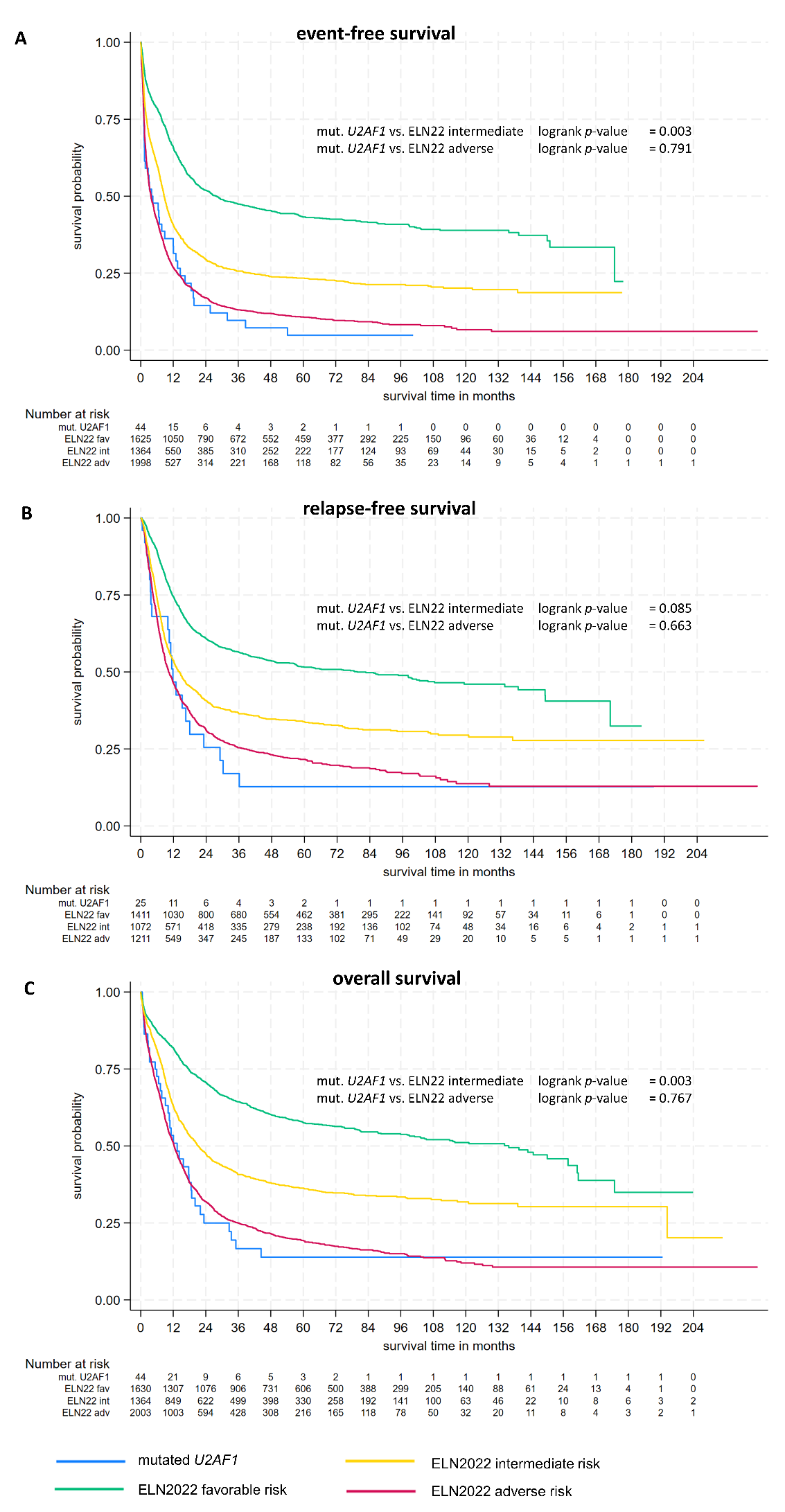


**Figure S7 Kaplan-Meier plots comparing ELN2022 risk groups to AML with altered *U2AF1*.** Event-free survival (EFS, A), relapse-free survival (RFS, B), and overall survival (OS, C) are shown. Patients from the entire cohort were retrospectively assigned to ELN2022 risk groups. Patients within the ELN2022 adverse risk group that had altered *U2AF1* were treated as a separate group for this Kaplan-Meier analysis. Patients with *U2AF1* and co-occurring class-defining favorable or intermediate risk features were treated as favorable or intermediate risk, respectively.


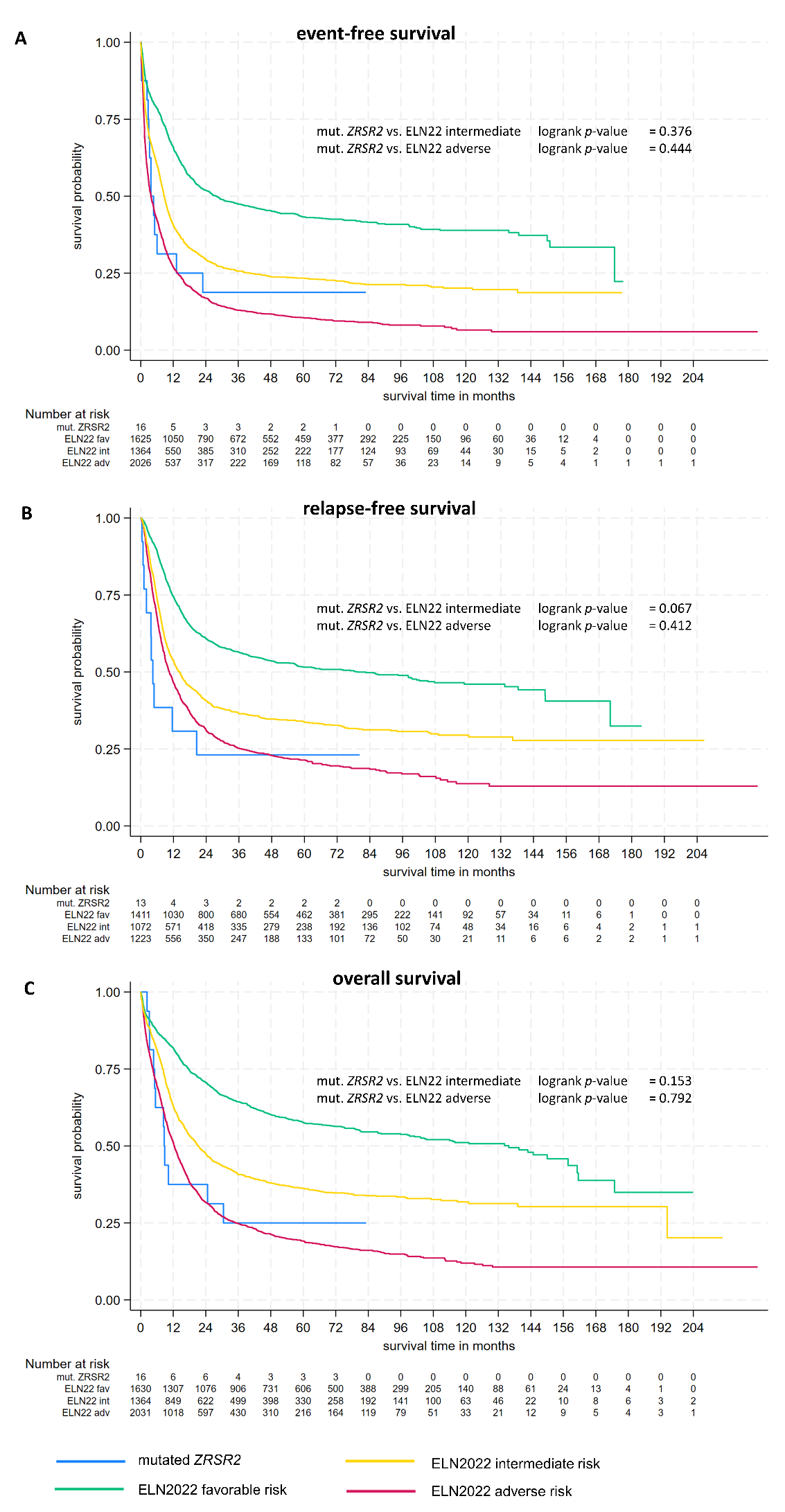


**Figure S8 Kaplan-Meier plots comparing ELN2022 risk groups to AML with altered *ZRSR2*.** Event-free survival (EFS, A), relapse-free survival (RFS, B), and overall survival (OS, C) are shown. Patients from the entire cohort were retrospectively assigned to ELN2022 risk groups. Patients within the ELN2022 adverse risk group that had altered *ZRSR2* were treated as a separate group for this Kaplan-Meier analysis. Patients with *ZRSR2* and co-occurring class-defining favorable or intermediate risk features were treated as favorable or intermediate risk, respectively.


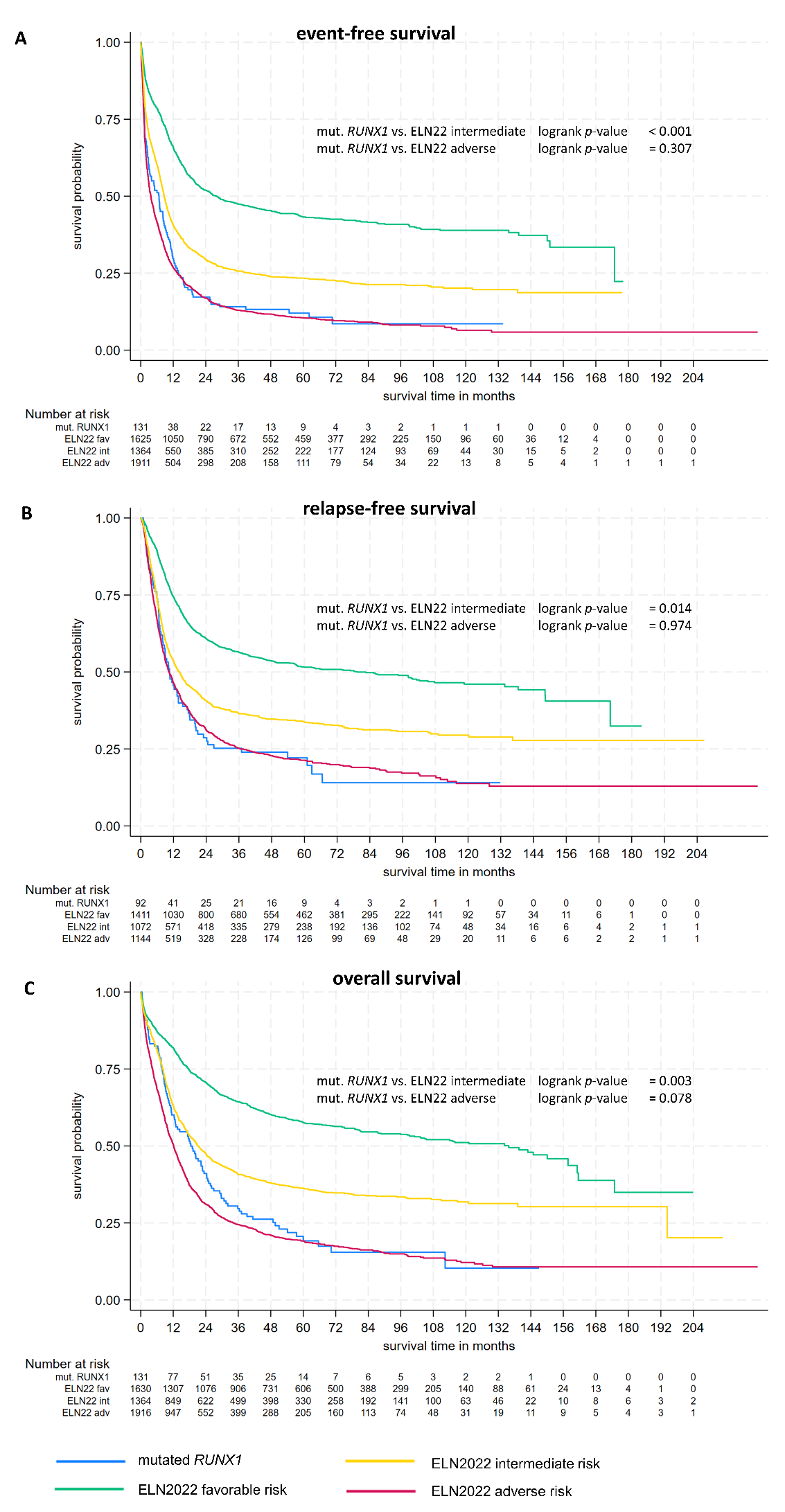


**Figure S9 Kaplan-Meier plots comparing ELN2022 risk groups to AML with altered *RUNX1*.** Event-free survival (EFS, A), relapse-free survival (RFS, B), and overall survival (OS, C) are shown. Patients from the entire cohort were retrospectively assigned to ELN2022 risk groups. Patients within the ELN2022 adverse risk group that had altered *ZRSR2* were treated as a separate group for this Kaplan-Meier analysis. Patients with *ZRSR2* and co-occurring class-defining favorable or intermediate risk features were treated as favorable or intermediate risk, respectively.
